# A novel adaptive quasi-Newton-type update and its global convergence without Lipschitz condition for constrained system of nonlinear monotone equations

**DOI:** 10.1371/journal.pone.0344697

**Published:** 2026-06-10

**Authors:** Kabiru Ahmed, Hatem E. Semary, Asmaa S. Al-Moisheer, Sulaiman M. Ibrahim, Abubakar Sani Halilu, Muhammed Yusuf Waziri, Mohamad Afendee Mohamed, Salisu Murtala

**Affiliations:** 1 Department of Mathematical Sciences, Bayero University, Kano, Nigeria; 2 Department of Mathematical Sciences, Federal University, Dutse, Nigeria; 3 Department of Mathematics and Statistics, Faculty of Science, Imam Mohammad Ibn Saud Islamic University (IMSIU), Riyadh, Saudi Arabia; 4 College of Applied and Health Sciences, A’Sharqiyah University, Ibra, Sultanate of Oman; 5 Faculty of Education and Arts, Sohar University, Sohar, Oman; 6 Faculty of Informatics and Computing, Universiti Sultan Zainal Abidin, Terengganu, Malaysia; University of Dhaka, BANGLADESH

## Abstract

This paper presents a new iterative method with a restart feature for solving constrained system of nonlinear monotone equations. The scheme, which is a double-parameter method, was initiated by considering a positive-definite adaptation of the quasi-Newton update proposed by Andrei (J. Comput. Appl. Math. 332, 26–44 (2018)) for unconstrained optimization. The two scaling parameters of the method are obtained by employing the measure function by Byrd and Nocedal (SIAM J. Numer. Anal. 26, 727–739 (1989)), which ensures that the condition number of the update is minimized. Another important attribute of the method is that its global convergence analysis is conducted without the Lipschitz assumption, which is a strong condition. Furthermore, the two parameters embedded in the scheme help in maintaining a balance in the distribution of the eigenvalues of its update matrix. The method converges globally regardless of the line search procedure employed. Numerical experiments with some methods in the literature show that the scheme is effective.

## 1 Introduction

The interest in this paper is solution of the constrained system of nonlinear equations


$$\begin{array}{c} F(\bar{x})=0,\;\bar{x}\in ℬ\subseteq {\mathbb{R}}^{n}, \end{array}$$ (1)

where ℬ is a nonempty closed convex set and *F* is a continuous vector-valued mapping, which is also monotone, namely, it satisfies


$$\begin{array}{c} (F(x)-F(y){)}^{T}(x-y)\geq 0,\;\forall x,y\in {\mathbb{R}}^{n}. \end{array}$$ (2)

Real life applications of (1) can be found in studies such as general equilibrium problems in [1,2], the control systems analyzed in [3], networking and communications [4], modelling and data estimation [5], and in compressed sensing problems [6–14]. Some of the methods employed in solving (1) include adaptive versions of Newton’s method and its enhanced version, the quasi-Newton (QN) schemes [15–18], the derivative-free methods [19–24], Levenberg-Marquardt method [25], trust-region method [26] as well as spectral projection methods [27,28]. Despite their fast convergence properties, adaptations of Newton and QN methods have not been widely studied and few of the schemes for solving (1) have been developed. A viable reason for this is the huge memory required at each iteration to store matrices generated by the methods. The classical Newton and QN methods were developed to solve the optimization problem


$$\begin{array}{c} \min_{x\in {\mathbb{R}}^{n}}f(x), \end{array}$$ (3)

with *f* being a smooth real-valued nonlinear function, with gradient g(x)=∇f(x). The sequence of iterates generated by these methods are obtained via the formula


$$\begin{array}{c} x_{k+1}=x_{k}+\alpha_{k}d_{k},\;k=0,1,2\ldots , \end{array}$$

where $\alpha_{k}$ represents a step-size that is usually calculated using a line search procedure, and $d_{k}\in R^{n}$ is search direction that is computed via


$$\begin{array}{c} G_{k}d_{k}=-\nabla f(x_{k}), \end{array}$$

in Newton’s method, or


$$\begin{array}{c} B_{k}d_{k}=-\nabla f(x_{k}), \end{array}$$ (4)

in QN method, where $G_{k}\in R^{n\times n}=\nabla^{2}f(x_{k})$ is a matrix known as the true Hessian or its approximation *B*_*k*_ in the QN method. The first QN update was developed by Davidon [29] with subsequent analysis by Fletcher and Powell [30], hence the name DFP after the three authors. The update is defined by


$$\begin{array}{c} H_{k+1}=H_{k}-\frac{H_{k}y_{k}y_{k}^{T}H_{k}}{y_{k}^{T}H_{k}y_{k}}+\frac{s_{k}s_{k}^{T}}{y_{k}^{T}s_{k}}, \end{array}$$ (5)

in which $y_{k}=g_{k+1}-g_{k}$, $s_{k}=x_{k+1}-x_{k}$. By setting *H*_*k*_ to be the identity matrix, the memoryless version of (5) is obtained, namely


$$\begin{array}{c} H_{k+1}=I_{k}-\frac{y_{k}y_{k}^{T}}{y_{k}^{T}y_{k}}+\frac{s_{k}s_{k}^{T}}{y_{k}^{T}s_{k}}. \end{array}$$

A DFP scheme with the update (5) exhibit some nice properties, which includes quadratic termination, hereditary property, and generating conjugate directions for a quadratic objective function and an exact line search procedure. It retains positive-definiteness for general nonlinear functions, when the curvature condition $s_{k}^{T}y_{k}>0$ is satisfied. Iterations of the scheme require $3n^{2}+𝒪(n)$ and it has super-linear rate of convergence. Also, for strictly convex functions under the exact line search rule, the DFP scheme converges globally [31]. Notwithstanding these attributes, numerical studies and computations conducted have shown that the DFP update is usually unstable. This is due to the loss of positive-definiteness along some iterative points as well as the distribution of its eigenvalues. The Broyden [32], Fletcher [33], Goldfarb [34], and Shanno [35] (BFGS) update is the most prominent QN scheme available. Being a member of the Broyden family, the BFGS update is given by


$$\begin{array}{c} B_{k+1}=B_{k}-\frac{B_{k}s_{k}s_{k}^{T}B_{k}}{s_{k}^{T}B_{k}s_{k}}+\frac{y_{k}y_{k}^{T}}{y_{k}^{T}s_{k}},\;k=0,1,2\ldots , \end{array}$$ (6)

where *s*_*k*_ and *y*_*k*_ remain as defined in (5). For the purpose of practical computations, search directions of the QN methods (4) are usually expressed in closed form as


$$\begin{array}{c} d_{k+1}=-H_{k+1}g_{k+1}, \end{array}$$ (7)

where $H_{k+1}=B_{k+1}^{-1}$ denotes the inverse approximation to the Hessian of the BFGS method. By applying the popular Woodbury formula [36] twice on (6), the inverse is obtained as


$$\begin{array}{c} H_{k+1}=H_{k}-\frac{s_{k}y_{k}^{T}H_{k}+H_{k}y_{k}s_{k}^{T}}{s_{k}^{T}y_{k}}+\left(1+\frac{y_{k}^{T}H_{k}y_{k}}{s_{k}^{T}y_{k}}\right)\frac{s_{k}s_{k}^{T}}{s_{k}^{T}y_{k}}. \end{array}$$ (8)

In order to avoid computing the matrix *H*_*k*_, which involves huge storage at each iteration, it is replaced with the identity matrix *I* and the so called memoryless update is obtained, i.e.,


$$\begin{array}{c} H_{k+1}=I-\frac{s_{k}y_{k}^{T}+y_{k}s_{k}^{T}}{s_{k}^{T}y_{k}}+\left(1+\frac{\|y_{k}{\|}^{2}}{s_{k}^{T}y_{k}}\right)\frac{s_{k}s_{k}^{T}}{s_{k}^{T}y_{k}}. \end{array}$$ (9)

Further analysis of (6) by Andrei [37] shows that efficiency of the BFGS method depends so much on structure of its eigenvalues. It was also shown by Powell [38] and Byrd et al. [39] that the BFGS method suffers more from large eigenvalues of (6) than small ones. By conducting some numerical experiments on the BFGS method, Gill and Leonard [40] discovered that large number of iterations or functions and gradient evaluations may be required on some problems for the scheme to converge. These shortcomings of the scheme were shown in [40] to mostly emanate from poor initial Hessian approximations or ill-conditioning along the iterations. To address these problems, some scaling procedures were applied on the update in (6). By scaling the third term in (6) with a positive parameter $\gamma_{k}$, Biggs [41] gave the modification


$$\begin{array}{c} B_{k+1}=B_{k}-\frac{B_{k}s_{k}s_{k}^{T}B_{k}}{s_{k}^{T}B_{k}s_{k}}+\gamma_{k}\frac{y_{k}y_{k}^{T}}{y_{k}^{T}s_{k}}. \end{array}$$

Oren and Luenberger [42] scaled the first two terms of the update in (6) to obtain


$$\begin{array}{c} B_{k+1}=\delta_{k}\left[B_{k}-\frac{B_{k}s_{k}s_{k}^{T}B_{k}}{s_{k}^{T}B_{k}s_{k}}\right]+\frac{y_{k}y_{k}^{T}}{y_{k}^{T}s_{k}}, \end{array}$$

where $\delta_{k}$ is a positive parameter. By adopting the technique of changing structure of eigenvalues Andrei [43] presented a two-parameter BFGS method, with *B*_*k*+1_ given as


$$\begin{array}{c} B_{k+1}=\delta_{k}\left[B_{k}-\frac{B_{k}s_{k}s_{k}^{T}B_{k}}{s_{k}^{T}B_{k}s_{k}}\right]+\gamma_{k}\frac{y_{k}y_{k}^{T}}{y_{k}^{T}s_{k}}, \end{array}$$

with $\delta_{k}$ and $\gamma_{k}$ being positive parameters. Here, $\delta_{k}$ is computed to ensure that eigenvalues of *B*_*k*+1_ are clustered, while $\gamma_{k}$ is obtained so that large eigenvalues of *B*_*k*+1_ are reduced, i.e., the eigenvalues are shifted to the left. This strategy brings about a better distribution of the eigenvalues. A modification of (9) was proposed in [44], namely


$$\begin{array}{c} H_{k+1}=\frac{1}{\delta_{k}}\left[H_{k}-\frac{H_{k}y_{k}s_{k}^{T}+s_{k}y_{k}^{T}H_{k}}{s_{k}^{T}y_{k}}+\left(\frac{\delta_{k}}{\gamma_{k}}+\frac{y_{k}^{T}H_{k}y_{k}}{s_{k}^{T}y_{k}}\right)\frac{s_{k}s_{k}^{T}}{s_{k}^{T}y_{k}}\right], \end{array}$$ (10)

where $\delta_{k}$ and $\gamma_{k}$ are parameters that are determined by employing Byrd and Nocedal’s measure function in [45]. The method proves to be computationally cheap and also has superlinear rate of convergence on practical problems.

In order to address some of the shortfalls of the DFP (5) and BFGS (6) updates and address some of their shortfalls, some variants for solving problem (3) have been proposed in the literature. Based on the $\ell_{\infty }$ matrix norm and an upper bound for condition number of the scaled version of (9), Babaie-Kafaki [46] proposed a BFGS-type method for the unconstrained optimization problem (3). The author proved that the method satisfy the descent condition and conducted numerical experiments to show its effectiveness. Also, Babaie-Kafaki and Aminifard [47] proposed two parameter scaled memoryless BFGS update in (8) for the problem (3) with a monotone choice for the initial step length. The authors proved the sufficient descent condition of the scheme for uniformly convex functions. Aminifard and Babaie-Kafaki [48] developed a DFP-type method for solving (3) by analyzing eigenvalues of a scaled memoryless variant of the DFP update (5), where the scaled parameter is obtained by skipping the direction of maximum magnification. By exploiting a spectral secant condition for the classical DFP update (5), Dargahi et al. [49] proposed a DFP-type method for (3), where the updating matrix is obtained such that its condition number is minimized. Bakhshinejad and Babaie-Kafaki [50] also proposed a DFP-type algorithm as an extension of the search direction obtained by applying the DFP update (5). The method was shown to satisfy the much required sufficient descent condition.

In order to exploit some of the advantages of the DFP and BFGS updates and address their shortcomings, some adaptive versions for solving (1) and its unconstrained form have been proposed in recent years. In line with this, Mamat et al. [51] proposed a DFP-type algorithm for solving the symmetric form of problem (1) by employing a scaled memoryless version of the DFP update (5). The authors proved global convergence of the scheme by employing some basic assumptions. As an enhancement of the classical DFP update (5), Dauda [52] proposed a three-term DFP-type method for solving the symmetric form of (1). The authors proved its global convergence using some mild conditions. Based on a scaled memoryless version of the DFP update (5), Awwal et al. [53] proposed a derivative-free method for solving (1). The authors proved global convergence of the method and compared its performance with some methods for solving (1). Inspired by a scaled version of the memoryless DFP update (5), another derivative-free method was proposed by Rao and Huang [54] for the unconstrained form of (1). Global convergence of the method was shown by employing some mild conditions. By applying a scaling technique and the measure function of Byrd and Nocedal [45] on a scaled memoryless DFP update (5), Ullah et al. [55] proposed a derivative-free DFP-type method for the constrained problem (1). Both global convergence and numerical efficiency were shown by the authors. In a recent development, Rehman et al. [56] proposed another version of a derivative-free DFP method for solving (1). The method converges globally and its effectiveness was illustrated by comparison with some recent methods in the literature. Recently, by computing an optimal value for the scaled memoryless BFGS update (9), Shah et al. [57] proposed a scaled derivative-free BFGS method for solving (1). The authors proved global convergence of the scheme and demonstrated its application to motion control. For more recent methods for solving (1), the reader can refer to works in [58–65]. The objectives of this paper are as follows:

- To develop a QN-type algorithm close to the BFGS method, for solving problem (1), which avoids the explicit computation of any matrix in the algorithm.
- To develop a scheme that exhibits some nice attributes of the classical BFGS update, addresses some of its shortcomings, and which possess the vital property for analyzing the convergence for problem (1).
- To develop a globally convergent algorithm without the Lipschitz condition and to analyze its numerical performance in solving problem (1).

The paper is structured as follows: Motivation and details of the method are given in the following section. Analysis of its global convergence is given in Section 3. Results of some numerical experiments of the scheme and some recent methods in the literature are presented in Section 4. Conclusions are given in Section 5.

## 2 An adaptive QN-type method

An adaptive QN-type scheme will be derived in this section. To achieve that, the following lemma is imperative:

**Lemma 2.1. *Cauchy Schwarz inequality***

*Let* $\overset{―}{V}$ *be an inner product space over the field of complex numbers* ℂ *with inner product* ⟨.,.⟩*. Then, for every pair of vectors* $x,y\in \overset{―}{V}$*, the following inequality holds.*


$$\begin{array}{c} |\langle x,y\rangle {|}^{2}\leq \langle x,x\rangle \langle y,y\rangle . \end{array}$$ (11)

As stated in the introduction section, adaptations of the iterative methods for the problem in (3) have been developed over the years for solving (1). However, to the best of our knowledge few QN-type schemes exists in the literature. The ones available include the derivative-free DFP-type method proposed by Awwal et al. [53], where the authors scaled the first term of the classical DFP update (5) with a positive parameter $\gamma_{k}$ to present


$$\begin{array}{c} H_{k+1}=\mu_{k}H_{k}-\frac{H_{k}y_{k}y_{k}^{T}H_{k}}{y_{k}^{T}H_{k}y_{k}}+\frac{s_{k}s_{k}^{T}}{y_{k}^{T}s_{k}},\;k=0,1,2\ldots , \end{array}$$ (12)

where $\mu_{k}$ represents a positive parameter that is computed such that the condition for analyzing global convergence holds for the scheme. Setting $H_{k}=\gamma_{k}I$ in (12) yields


$$\begin{array}{c} H_{k+1}=\mu_{k}\gamma_{k}I-\frac{\gamma_{k}y_{k}y_{k}^{T}}{y_{k}^{T}y_{k}}+\frac{s_{k}s_{k}^{T}}{y_{k}^{T}s_{k}},\;k=0,1,2\ldots , \end{array}$$ (13)

where $\gamma_{k}\in [{\bar{\gamma }}_{1},{\bar{\gamma }}_{2}]$, ${\bar{\gamma }}_{1},{\bar{\gamma }}_{2}>0$ is a scaling parameter. By substituting (13) into (7), the authors obtained the search direction


$$\begin{array}{c} d_{k+1}=-\mu_{k}\gamma_{k}F_{k+1}-\frac{s_{k}^{T}F_{k+1}}{s_{k}^{T}y_{k}}s_{k}+\gamma_{k}\frac{y_{k}^{T}F_{k+1}}{y_{k}^{T}y_{k}}y_{k},\;k=0,1,2\ldots , \end{array}$$

with $\mu_{k}\geq \alpha +1$, α>0. The choice of $\mu_{k}$ suggested by the authors only guarantees the condition for analyzing global convergence holds, it does not ensure stability of the proposed method. Following the work by Awwal et al. [53], Ullah et al. [55] scaled the third term of the memoryless version of the DFP update (5) with a positive parameter $\gamma_{k}$ to obtain


$$\begin{array}{c} H_{k+1}=I_{n}-\frac{y_{k}y_{k}^{T}}{y_{k}^{T}y_{k}}+\gamma_{k}\frac{s_{k}s_{k}^{T}}{y_{k}^{T}s_{k}},\;k=0,1,2\ldots , \end{array}$$ (14)

where $\gamma_{k}$,which is derived by employing the measure function of Nocedal and Byrd [45] is obtained as $\frac{s_{k}^{T}y_{k}}{\|s_{k}{\|}^{2}}$. Using this value of $\gamma_{k}$, the authors presented search direction of the scheme as


$$\begin{array}{c} d_{k+1}=-F_{k+1}+\frac{F_{k+1}^{T}y_{k}}{\|y_{k}{\|}^{2}}y_{k}-\frac{F_{k+1}^{T}s_{k}}{\|s_{k}{\|}^{2}}s_{k}. \end{array}$$ (15)

The value of the scaling parameter $\gamma_{k}$ obtained reduces the chances of the DFP updating matrix from being ill-conditioned. Still, it does not ensure that the search direction (15) satisfies the condition for analyzing global convergence. Following this, Shah et. al. [57] proposed another QN-type method for solving (1) by scaling the first and second term of the memoryless version of the Hessian approximation of the BFGS update [66], namely


$$\begin{array}{c} B_{k+1}=\gamma_{k}I-\gamma_{k}\frac{s_{k}s_{k}^{T}}{s_{k}^{T}s_{k}}+\frac{y_{k}y_{k}^{T}}{y_{k}^{T}s_{k}},\;k=0,1,2\ldots . \end{array}$$ (16)

The authors minimized the condition number of (16) to obtain the choice of $\gamma_{k}$ as $\frac{\left\|y_{k}\right\|}{\left\|s_{k}\right\|}$ resulting in the search direction


$$\begin{array}{c} d_{k+1}=-\frac{\left\|s_{k}\right\|}{\left\|y_{k}\right\|}F_{k+1}+\left(\frac{s_{k}^{T}F_{k+1}y_{k}+y_{k}^{T}F_{k+1}s_{k}}{y_{k}^{T}s_{k}}\right)\frac{\left\|s_{k}\right\|}{\left\|y_{k}\right\|}-\frac{\|y_{k}{\|}^{2}s_{k}^{T}F_{k+1}s_{k}}{(y_{k}^{T}s_{k}{)}^{2}}\frac{\left\|s_{k}\right\|}{\left\|y_{k}\right\|}-\frac{s_{k}^{T}F_{k+1}s_{k}}{y_{k}^{T}s_{k}}. \end{array}$$

In (16), the parameter $\gamma_{k}$ does not always ensure stability of the BFGS method as the third term on the right may grow larger producing a shift of the eigenvalues to the right. In a recent attempt to address the shortfalls of the DFP update (5), Rehman et. al. [56] scaled the first and second terms of the memoryless DFP update (5), namely


$$\begin{array}{c} H_{k+1}=\gamma_{k}I_{n}-\gamma_{k}\frac{y_{k}y_{k}^{T}}{y_{k}^{T}y_{k}}+\frac{s_{k}s_{k}^{T}}{y_{k}^{T}s_{k}},\;k=0,1,2\ldots , \end{array}$$ (17)

with $\gamma_{k}$ being a positive parameter whose optimal value was obtained as the minimizer of the condition number of *H*_*k*+1_ which is given as


$$\begin{array}{c} \gamma_{k}=\frac{\left\|s_{k}\right\|}{\left\|y_{k}\right\|}. \end{array}$$ (18)

By substituting (18) into the memoryless DFP update (17), the authors obtained


$$\begin{array}{c} H_{k+1}=\frac{\left\|s_{k}\right\|}{\left\|y_{k}\right\|}I_{n}-\frac{\left\|s_{k}\right\|}{\left\|y_{k}\right\|}\frac{y_{k}y_{k}^{T}}{y_{k}^{T}y_{k}}+\frac{s_{k}s_{k}^{T}}{y_{k}^{T}s_{k}},\;k=0,1,2\ldots , \end{array}$$ (19)

and the search direction


$$\begin{array}{c} d_{k+1}=-\frac{\left\|s_{k}\right\|}{\left\|y_{k}\right\|}F_{k+1}+\frac{\left\|s_{k}\right\|}{\left\|y_{k}\right\|}\frac{F_{k+1}^{T}y_{k}}{\|y_{k}{\|}^{2}}y_{k}-\frac{F_{k+1}^{T}s_{k}}{s_{k}^{T}y_{k}}s_{k}. \end{array}$$

Now, trace of the matrix *H*_*k*+1_ in (19) can be computed as


$$\begin{array}{c} \mathrm{tr}(H_{k+1})=\frac{\left\|s_{k}\right\|}{\left\|y_{k}\right\|}n-\frac{\left\|s_{k}\right\|}{\left\|y_{k}\right\|}+\frac{\|s_{k}{\|}^{2}}{s_{k}^{T}y_{k}}. \end{array}$$ (20)

It can be seen that the third-term on the right of (20) will shift the eigenvalues of *H*_*k*+1_ towards the right making the matrix to have large eigenvalues. On the other hand, the second term being negative tends to shift the eigenvalues to the left. Even though this effect is countered by the first term, which is positive, it does not ensure proper distribution of the eigenvalues of *H*_*k*+1_, and by extension stability of the scheme is not guaranteed.

**Remark 2.2** *As explained above, while the choices of the parameters* $\mu_{k}$ *and* $\gamma_{k}$ *in* (13), (14), (16), and (17) may ena*ble the scheme generated with the corresponding matrix* *H*_*k*+1_ *to satisfy the vital condition for analyzing global convergence, they do not ensure its stability – a condition attributed to uneven distribution of eigenvalues of* *H*_*k*+1_*. In addition to this, like most of the published works in the literature for the constrained problem (1), the methods in [53–56] depend on the Lipschitz condition for proving their global convergence. And since not all problems satisfy the Lipschitz condition, the number of problems analyzed tend to be limited.*

Now, by replacing *s*_*k*_ with ${\bar{y}}_{k-1}$, *y*_*k*_ with *F*_*k*−1_, *B*_*k*+1_ with *Q*_*k*_ in (6), and motivated by the scaling strategy in (10) and the above remark, we present the following two parameter scaled symmetric rank-two quasi-Newton type update,


$$\begin{array}{c} Q_{k}=\frac{1}{\gamma_{k-1}}\left[Q_{k-1}-\frac{Q_{k-1}{\bar{y}}_{k-1}{\bar{y}}_{k-1}^{T}Q_{k-1}}{{\bar{y}}_{k-1}^{T}Q_{k-1}{\bar{y}}_{k-1}}\right]+\frac{1}{\delta_{k-1}}\frac{F_{k-1}F_{k-1}^{T}}{F_{k-1}^{T}{\bar{y}}_{k-1}}, \end{array}$$ (21)

in which $F_{k-1}=F(x_{k-1})$ and


$$\begin{array}{c} {\bar{y}}_{k-1}=F(\psi_{k-1})-F(x_{k-1})+\nu s_{k-1},\;\psi_{k-1}=x_{k-1}+\alpha_{k-1}d_{k-1},\;\nu >0. \end{array}$$ (22)

Setting *Q*_*k*−1_ as the identity matrix yields the following scaled memoryless version of (21):


$$\begin{array}{c} Q_{k}=\frac{1}{\gamma_{k-1}}\left[I-\frac{{\bar{y}}_{k-1}{\bar{y}}_{k-1}^{T}}{{\bar{y}}_{k-1}^{T}{\bar{y}}_{k-1}}\right]+\frac{1}{\delta_{k-1}}\frac{F_{k-1}F_{k-1}^{T}}{F_{k-1}^{T}{\bar{y}}_{k-1}}. \end{array}$$ (23)

Now, in order to define search direction of the proposed scheme, we require the inverse of the update *Q*_*k*_ and appropriate values for the positive parameters $\gamma_{k-1}$ and $\delta_{k-1}$. To achieve that goal, we first show that *Q*_*k*_ is positive-definite. Assuming that $F_{k-1}^{T}{\bar{y}}_{k-1}\geq m\|F_{k-1}\|\|{\bar{y}}_{k-1}\|>0$, with m∈(0,1], then we have that $F_{k-1}\neq 0$ and ${\bar{y}}_{k-1}\neq 0$. This further implies that a set of mutually orthonormal vectors $v_{k-1}^{1},v_{k-1}^{2},...v_{k-1}^{n-2}\in {\mathbb{R}}^{n}$ exists such that $F_{k-1}^{T}v_{k-1}^{i}={\bar{y}}_{k-1}^{T}v_{k-1}^{i}=0$, i=1,...,n−2, which determines that


$$\begin{array}{c} Q_{k}v_{k-1}^{i}=Q_{k}^{T}v_{k-1}^{i}=\frac{1}{\gamma_{k-1}}v_{k-1}^{i},\;i=1,...,n-2. \end{array}$$

Thus, *Q*_*k*_ contains *n* − 2 eigenvalues equal to $\frac{1}{\gamma_{k-1}}$ each. We label the two eigenvalues left as $\zeta_{k-1}^{+}$ and $\zeta_{k-1}^{-}$.

From (23), the trace of *Q*_*k*_ is obtained as


$$\begin{array}{cc} \mathrm{tr}(Q_{k}) & =\frac{n}{\gamma_{k-1}}-\frac{1}{\gamma_{k-1}}+\frac{\|F_{k-1}{\|}^{2}}{\delta_{k-1}F_{k-1}^{T}{\bar{y}}_{k-1}}\\ & =\underset{\text{(n-2) times}}{\underbrace{\frac{1}{\gamma_{k-1}}+...+\frac{1}{\gamma_{k-1}}}}+\zeta_{k-1}^{+}+\zeta_{k-1}^{-}, \end{array}$$ (24)

which then reveals that


$$\begin{array}{c} \zeta_{k-1}^{+}+\zeta_{k-1}^{-}=\frac{1}{\gamma_{k-1}}+\frac{\|F_{k-1}{\|}^{2}}{\delta_{k-1}F_{k-1}^{T}{\bar{y}}_{k-1}}. \end{array}$$ (25)

Applying the Sherman-Morrison formula [36], the inverse of *Q*_*k*_ is computed as


$$\begin{array}{c} Q_{k}^{-1}=\gamma_{k-1}I+\frac{(\delta_{k-1}F_{k-1}^{T}{\bar{y}}_{k-1}+\gamma_{k-1}\|F_{k-1}{\|}^{2}){\bar{y}}_{k-1}{\bar{y}}_{k-1}^{T}}{(F_{k-1}^{T}{\bar{y}}_{k-1}{)}^{2}}-\frac{\gamma_{k-1}{\bar{y}}_{k-1}F_{k-1}^{T}}{F_{k-1}^{T}{\bar{y}}_{k-1}}-\frac{\gamma_{k-1}F_{k-1}{\bar{y}}_{k-1}^{T}}{F_{k-1}^{T}{\bar{y}}_{k-1}}. \end{array}$$ (26)

Since $Q_{k}^{-1}$ has *n* − 2 repeated eigenvalues equal to $\gamma_{k-1}$ each, labelling the remaining two as $\frac{1}{\zeta_{k-1}^{+}}$ and $\frac{1}{\zeta_{k-1}^{-}}$, we obtain from (26) that


$$\begin{array}{c} det(Q_{k}^{-1})=\gamma_{k-1}^{\left(n-2\right)}\frac{1}{\zeta_{k-1}^{+}\zeta_{k-1}^{-}}. \end{array}$$ (27)

Also, expressing $Q_{k}^{-1}$ as the product of two matrices yields


$$\begin{array}{c} Q_{k}^{-1}=\gamma_{k-1}I\left(I+\frac{(\delta_{k-1}F_{k-1}^{T}{\bar{y}}_{k-1}+\gamma_{k-1}\|F_{k-1}{\|}^{2}){\bar{y}}_{k-1}{\bar{y}}_{k-1}^{T}}{\gamma_{k-1}(F_{k-1}^{T}{\bar{y}}_{k-1}{)}^{2}}-\frac{{\bar{y}}_{k-1}F_{k-1}^{T}}{F_{k-1}^{T}{\bar{y}}_{k-1}}-\frac{F_{k-1}{\bar{y}}_{k-1}^{T}}{F_{k-1}^{T}{\bar{y}}_{k-1}}\right). \end{array}$$

Let $A=\gamma_{k-1}I$ and $B=I+\frac{((\delta_{k-1}F_{k-1}^{T}{\bar{y}}_{k-1}+\gamma_{k-1}\|F_{k-1}{\|}^{2}){\bar{y}}_{k-1}-\gamma_{k-1}(F_{k-1}^{T}{\bar{y}}_{k-1})F_{k-1}){\bar{y}}_{k-1}}{\gamma_{k-1}(F_{k-1}^{T}{\bar{y}}_{k-1}{)}^{2}}$, then


$$\begin{array}{c} det(A)=\gamma_{k-1}^{n}. \end{array}$$ (28)

By applying the formula for determinant of an arbitrary matrix of rank two, namely


$$\begin{array}{c} det(I+a_{1}a_{2}^{T}+a_{3}a_{4}^{T})=(1+a_{1}^{T}a_{2})(1+a_{3}^{T}a_{4})-(a_{1}^{T}a_{4})(a_{2}^{T}a_{3}), \end{array}$$

and setting $a_{1}=-\frac{{\bar{y}}_{k-1}}{F_{k-1}^{T}{\bar{y}}_{k-1}}$, $a_{2}=F_{k-1}$, $a_{3}=\frac{(\delta_{k-1}F_{k-1}^{T}{\bar{y}}_{k-1}+\gamma_{k-1}\|F_{k-1}{\|}^{2}){\bar{y}}_{k-1}-\gamma_{k-1}(F_{k-1}^{T}{\bar{y}}_{k-1})F_{k-1}}{\gamma_{k-1}(F_{k-1}^{T}{\bar{y}}_{k-1}{)}^{2}}$, and $a_{4}={\bar{y}}_{k-1}$, we obtain


$$\begin{array}{c} det(B)=\frac{\delta_{k-1}\|{\bar{y}}_{k-1}{\|}^{2}}{\gamma_{k-1}F_{k-1}^{T}{\bar{y}}_{k-1}}. \end{array}$$ (29)

Therefore, from (28) and (29), we obtain


$$\begin{array}{c} det(Q_{k}^{-1})=\gamma_{k-1}^{\left(n-1\right)}\frac{\delta_{k-1}\|{\bar{y}}_{k-1}{\|}^{2}}{F_{k-1}^{T}{\bar{y}}_{k-1}}. \end{array}$$ (30)

Now, combining (27), (30) with some simplification, we get


$$\begin{array}{c} \zeta_{k-1}^{+}\zeta_{k-1}^{-}=\frac{F_{k-1}^{T}{\bar{y}}_{k-1}}{\gamma_{k-1}\delta_{k-1}\|{\bar{y}}_{k-1}{\|}^{2}}. \end{array}$$ (31)

Using (25) and (31), we obtain


$$\begin{array}{c} \zeta_{k-1}^{\pm }=\frac{1}{2}\left[\frac{1}{\gamma_{k-1}}+\frac{\|F_{k-1}{\|}^{2}}{\delta_{k-1}F_{k-1}^{T}{\bar{y}}_{k-1}}\pm \sqrt{{\left(\frac{1}{\gamma_{k-1}}+\frac{\|F_{k-1}{\|}^{2}}{\delta_{k-1}F_{k-1}^{T}{\bar{y}}_{k-1}}\right)}^{2}-4\frac{F_{k-1}^{T}{\bar{y}}_{k-1}}{\gamma_{k-1}\delta_{k-1}\|{\bar{y}}_{k-1}{\|}^{2}}}\right], \end{array}$$

which can also be expressed as


$$\begin{array}{cc} \zeta_{k-1}^{\pm } & =\frac{1}{2}[\frac{1}{\gamma_{k-1}}+\frac{\|F_{k-1}{\|}^{2}}{\delta_{k-1}F_{k-1}^{T}{\bar{y}}_{k-1}}\\ & \pm \sqrt{{\left(\frac{1}{\gamma_{k-1}}-\frac{\|F_{k-1}{\|}^{2}}{\delta_{k-1}F_{k-1}^{T}{\bar{y}}_{k-1}}\right)}^{2}+4\frac{\|F_{k-1}{\|}^{2}}{\gamma_{k-1}\delta_{k-1}F_{k-1}^{T}{\bar{y}}_{k-1}}-4\frac{F_{k-1}^{T}{\bar{y}}_{k-1}}{\gamma_{k-1}\delta_{k-1}\|{\bar{y}}_{k-1}{\|}^{2}}}]. \end{array}$$ (32)

Now, by applying the inequality in (11) to (32), we obtain


$$\begin{array}{cc} \zeta_{k-1}^{+} & \geq \frac{1}{2}\left[\frac{1}{\gamma_{k-1}}+\frac{\|F_{k-1}{\|}^{2}}{\delta_{k-1}F_{k-1}^{T}{\bar{y}}_{k-1}}+\sqrt{{\left(\frac{1}{\gamma_{k-1}}-\frac{\|F_{k-1}{\|}^{2}}{\delta_{k-1}F_{k-1}^{T}{\bar{y}}_{k-1}}\right)}^{2}}\right]\\ & =\frac{1}{2}\left[\frac{1}{\gamma_{k-1}}+\frac{\|F_{k-1}{\|}^{2}}{\delta_{k-1}F_{k-1}^{T}{\bar{y}}_{k-1}}+\frac{1}{\gamma_{k-1}}-\frac{\|F_{k-1}{\|}^{2}}{\delta_{k-1}F_{k-1}^{T}{\bar{y}}_{k-1}}\right]\\ & =\frac{1}{\gamma_{k-1}}. \end{array}$$ (33)

Also, by employing the Cauchy Schwarz inequality in (11) and applying similar argument, we have that $0<\zeta_{k-1}^{-}\leq \frac{1}{\gamma_{k-1}}$, which applying (33) consequently yields


$$\begin{array}{c} 0<\zeta_{k-1}^{-}\leq \frac{1}{\gamma_{k-1}}\leq \zeta_{k-1}^{+}. \end{array}$$ (34)

Following (34), and since $\gamma_{k-1}$ is a positive parameter, we conclude that the matrix *Q*_*k*_ is positive-definite, which implies that $Q_{k}^{-1}$ is also positive-definite. We are now set to employ the measure function of Byrd and Nocedal [45] to compute approximations of $\gamma_{k-1}$ and $\delta_{k-1}$ that will ensure well-conditioning of the matrix *Q*_*k*_ as well as better distribution of its eigenvalues. The measure function is formulated as


$$\begin{array}{c} \phi (Q_{k})=\mathrm{tr}(Q_{k})-\ln(det(Q_{k})). \end{array}$$ (35)

From (23) and applying the same approach employed to compute the determinant of $Q_{k}^{-1}$ earlier, we obtain the determinant of *Q*_*k*_ as


$$\begin{array}{c} det(Q_{k})=\frac{\gamma_{k-1}^{\left(1-n\right)}}{\delta_{k-1}}\frac{F_{k-1}^{T}{\bar{y}}_{k-1}}{\|{\bar{y}}_{k-1}{\|}^{2}}. \end{array}$$ (36)

Now, from (24), (35) and (36), we have


$$\begin{array}{cc} \phi (Q_{k}) & =\frac{n}{\gamma_{k-1}}-\frac{1}{\gamma_{k-1}}+\frac{\|F_{k-1}{\|}^{2}}{\delta_{k-1}F_{k-1}^{T}{\bar{y}}_{k-1}}-\ln(\frac{\gamma_{k-1}^{\left(1-n\right)}}{\delta_{k-1}}\frac{F_{k-1}^{T}{\bar{y}}_{k-1}}{\|{\bar{y}}_{k-1}{\|}^{2}})\\ & =\frac{n}{\gamma_{k-1}}-\frac{1}{\gamma_{k-1}}+\frac{\|F_{k-1}{\|}^{2}}{\delta_{k-1}F_{k-1}^{T}{\bar{y}}_{k-1}}-\ln(\gamma_{k-1}^{\left(1-n\right)})+\ln(\delta_{k-1})-\ln(F_{k-1}^{T}{\bar{y}}_{k-1})+\ln(\|{\bar{y}}_{k-1}{\|}^{2})\\ & =\frac{n}{\gamma_{k-1}}-\frac{1}{\gamma_{k-1}}+\frac{\|F_{k-1}{\|}^{2}}{\delta_{k-1}F_{k-1}^{T}{\bar{y}}_{k-1}}-(1-n)\ln(\gamma_{k-1})+\ln(\delta_{k-1})-\ln(F_{k-1}^{T}{\bar{y}}_{k-1})\\ & +\ln(\|{\bar{y}}_{k-1}{\|}^{2}). \end{array}$$ (37)

In order to obtain approximations for $\gamma_{k-1}$ and $\delta_{k-1}$, we have to minimize the function $\phi (Q_{k})$ with respect to both $\gamma_{k-1}$ and $\delta_{k-1}$. So, from (37), we get


$$\begin{array}{c} \frac{d\phi (Q_{k})}{d\gamma_{k-1}}=\frac{1}{\gamma_{k-1}^{2}}-\frac{n}{\gamma_{k-1}^{2}}-\frac{\left(1-n\right)}{\gamma_{k-1}}. \end{array}$$

Setting this result to zero, yields


$$\begin{array}{c} \frac{1}{\gamma_{k-1}^{2}}-\frac{n}{\gamma_{k-1}^{2}}-\frac{\left(1-n\right)}{\gamma_{k-1}}=0, \end{array}$$

which implies that


$$\begin{array}{c} \gamma_{k-1}(1-n)=1-n, \end{array}$$

or


$$\begin{array}{c} \gamma_{k-1}=1. \end{array}$$ (38)

Also,


$$\begin{array}{c} \frac{d\phi (Q_{k})}{d\delta_{k-1}}=\frac{1}{\delta_{k-1}}-\frac{1}{\delta_{k-1}^{2}}\frac{\|F_{k-1}{\|}^{2}}{F_{k-1}^{T}{\bar{y}}_{k-1}}. \end{array}$$

Setting this result to zero yields


$$\begin{array}{c} \frac{1}{\delta_{k-1}}-\frac{1}{\delta_{k-1}^{2}}\frac{\|F_{k-1}{\|}^{2}}{F_{k-1}^{T}{\bar{y}}_{k-1}}=0, \end{array}$$

which implies that


$$\begin{array}{c} \frac{1}{\delta_{k-1}^{2}}\frac{\|F_{k-1}{\|}^{2}}{F_{k-1}^{T}{\bar{y}}_{k-1}}=\frac{1}{\delta_{k-1}}, \end{array}$$

or


$$\begin{array}{c} \delta_{k-1}=\frac{\|F_{k-1}{\|}^{2}}{F_{k-1}^{T}{\bar{y}}_{k-1}}. \end{array}$$ (39)

Following the above analysis and the fact that restarting search directions tend to improve convergence and efficiency of algorithms [67], we substitute (38) and (39) into (26), and propose the following auxiliary search direction:


$$\begin{array}{c} d_{k}=\left\{ \begin{array}{c} -F_{k},\;k=0,\\ -F_{k}+\frac{F_{k}^{T}F_{k-1}}{F_{k-1}^{T}{\bar{y}}_{k-1}}{\bar{y}}_{k-1}+\frac{F_{k}^{T}{\bar{y}}_{k-1}}{F_{k-1}^{T}{\bar{y}}_{k-1}}F_{k-1}-2\frac{\|F_{k-1}{\|}^{2}F_{k}^{T}{\bar{y}}_{k-1}}{(F_{k-1}^{T}{\bar{y}}_{k-1}{)}^{2}}{\bar{y}}_{k-1},\text{ for }k\geq 1\text{ and}\\ F_{k-1}^{T}{\bar{y}}_{k-1}\geq m\|F_{k-1}\|\|{\bar{y}}_{k-1}\|,\;m\in (0,1],\\ -F_{k}+\frac{1}{r}\frac{F_{k}^{T}{\bar{y}}_{k-1}}{{\bar{y}}_{k-1}^{T}{\bar{y}}_{k-1}}{\bar{y}}_{k-1}-\frac{\left|F_{k-1}^{T}{\bar{y}}_{k-1}\right|}{\left\|F_{k-1}\|\|{\bar{y}}_{k-1}\right\|}\frac{F_{k}^{T}F_{k-1}}{\|F_{k-1}{\|}^{2}}F_{k-1},\;r>1,\text{ otherwise}. \end{array}\right. \end{array}$$ (40)

**Remark 2.3** *It should be noted that the inequality* $F_{k-1}^{T}{\bar{y}}_{k-1}\geq m\|F_{k-1}\|\|{\bar{y}}_{k-1}\|$*, which triggers the restart process was required for two reasons: one, it is essential for analyzing eigenvalues of the matrix* *Q*_*k*_*, and two, it helps in proving boundedness of the search direction.*

**Lemma 2.4** *The search direction* *d*_*k*_ *in (*40) sa*tisfies the inequality*


$$\begin{array}{c} d_{k}^{T}F_{k}\leq c\|F_{k}{\|}^{2},\;c=\min\left\{\left(1-\frac{1}{r}\right),\frac{1}{2}\right\},\;\forall k\geq 1. \end{array}$$ (41)

**Proof**: First, from (40) for *k* = 0, we have


$$\begin{array}{c} d_{0}^{T}F_{0}=-F_{0}^{T}(F_{0})=-\|F_{0}{\|}^{2}. \end{array}$$

Now, we proceed to analyze each of the two cases in (40) for *k* ≥ 1.

1^*st*^ Case: If $F_{k-1}^{T}{\bar{y}}_{k-1}\geq m\|F_{k-1}\|\|{\bar{y}}_{k-1}\|$, we have


$$\begin{array}{cc} d_{k}^{T}F_{k} & =-\|F_{k}{\|}^{2}+2\frac{(F_{k}^{T}F_{k-1})F_{k}^{T}{\bar{y}}_{k-1}}{F_{k-1}^{T}{\bar{y}}_{k-1}}-2\frac{\|F_{k-1}{\|}^{2}(F_{k}^{T}{\bar{y}}_{k-1}{)}^{2}}{(F_{k-1}^{T}{\bar{y}}_{k-1}{)}^{2}}\\ & =\frac{2F_{k}^{T}F_{k-1}(F_{k-1}^{T}{\bar{y}}_{k-1})F_{k}^{T}{\bar{y}}_{k-1}-(F_{k-1}^{T}{\bar{y}}_{k-1}{)}^{2}\|F_{k}{\|}^{2}-2\|F_{k-1}{\|}^{2}(F_{k}^{T}{\bar{y}}_{k-1}{)}^{2}}{(F_{k-1}^{T}{\bar{y}}_{k-1}{)}^{2}}\\ & \leq \frac{\frac{\|F_{k}{\|}^{2}(F_{k-1}^{T}{\bar{y}}_{k-1}{)}^{2}}{2}+2\|F_{k-1}{\|}^{2}(F_{k}^{T}{\bar{y}}_{k-1}{)}^{2}-\|F_{k}{\|}^{2}(F_{k-1}^{T}{\bar{y}}_{k-1}{)}^{2}-2\|F_{k-1}{\|}^{2}(F_{k}^{T}{\bar{y}}_{k-1}{)}^{2}}{(F_{k-1}^{T}{\bar{y}}_{k-1}{)}^{2}}\\ & =-\|F_{k}{\|}^{2}+\frac{\|F_{k}{\|}^{2}}{2}\\ & =-\left(1-\frac{1}{2}\right)\|F_{k}{\|}^{2}\\ & =-\frac{1}{2}\|F_{k}{\|}^{2}. \end{array}$$

2^*nd*^ Case: If $F_{k-1}^{T}{\bar{y}}_{k-1}<m\|F_{k-1}\|\|{\bar{y}}_{k-1}\|$, then from (40) and Cauchy Schwarz inequality, we get


$$\begin{array}{cc} d_{k}^{T}F_{k} & =-\|F_{k}{\|}^{2}+\frac{1}{r}\frac{(F_{k}^{T}{\bar{y}}_{k-1}{)}^{2}}{{\bar{y}}_{k-1}^{T}{\bar{y}}_{k-1}}-\frac{\left|F_{k-1}^{T}{\bar{y}}_{k-1}\right|}{\left\|F_{k-1}\|\|{\bar{y}}_{k-1}\right\|}\frac{(F_{k}^{T}F_{k-1}{)}^{2}}{\|F_{k-1}{\|}^{2}}\\ & \leq -\|F_{k}{\|}^{2}+\frac{1}{r}\frac{(F_{k}^{T}{\bar{y}}_{k-1}{)}^{2}}{{\bar{y}}_{k-1}^{T}{\bar{y}}_{k-1}}\\ & \leq -\|F_{k}{\|}^{2}+\frac{1}{r}\frac{\|F_{k}{\|}^{2}\|{\bar{y}}_{k-1}{\|}^{2}}{\|{\bar{y}}_{k-1}{\|}^{2}}\\ & =-\|F_{k}{\|}^{2}+\frac{1}{r}\|F_{k}{\|}^{2}\\ & =-\left(1-\frac{1}{r}\right)\|F_{k}{\|}^{2}. \end{array}$$

By setting $c=\min\left\{\left(1-\frac{1}{r}\right),\frac{1}{2}\right\}$, we established the proof.■

For the line search strategy, we employ the adaptive procedure proposed by Yin et. al. [68], where the step-size $\alpha_{k}$ is defined as $\alpha_{k}=\beta \rho^{i_{k}}$, with *i*_*k*_ being the smallest nonnegative integer *i* for which


$$\begin{array}{c} -F(\psi_{k}{)}^{T}d_{k}\geq \sigma \alpha_{k}P_{\left[\lambda ,u\right]}[\|F(\psi_{k})\|]\|d_{k}{\|}^{2}, \end{array}$$ (42)

for each $u\in {\mathbb{R}}^{n}$, $P_{\left[\lambda ,u\right]}[.]$ is the projection operator defined as


$$\begin{array}{c} P_{\left[\lambda ,u\right]}[h]=\left\{ \begin{array}{c} u,\text{ if }h\geq u,\\ \lambda ,\text{ if }h\leq \lambda ,\\ h,\text{ otherwise}, \end{array}\right. \end{array}$$

where 0<λ≤u, and *u* > 0.

Now, we described algorithm of the method as follows:

**Algorithm 1 (MKAS)**

**1**: *Initialization*. Select $x_{0}\in ℬ$, σ>0, ρ(0,1), m∈(0,1], *r* > 1, β∈(0,1], ϑ∈(0,2).

Set $d_{0}=-F_{0}$, *k*=0.

**2**: For $\|F_{k}\|\leq 10^{-10}$, stop. Else, move to **3**.

**3**: Compute $\psi_{k}$ as in (22), with $\alpha_{k}$ as defined earlier such that (42) holds.

**4**: If $\psi_{k}\in ℬ$ and $\|F(\psi_{k})\|\leq 10^{-10}$, stop $x_{k+1}=\psi_{k}$. Otherwise, compute


$$\begin{array}{c} x_{k+1}=P_{ℬ}\left[x_{k}-\vartheta \varsigma_{k}F(\psi_{k})\right],\text{ where} \end{array}$$ (43)

$P_{ℬ}(.)$ is the projection operator, which is defined in [69] with the property


$$\begin{array}{c} \|P_{ℬ}(x)-y\|\leq \|x-y\|,\;\forall y\in ℬ, \end{array}$$ (44)

with


$$\begin{array}{c} \varsigma_{k}=\frac{F(\psi_{k}{)}^{T}(x_{k}-\psi_{k})}{\|F(\psi_{k}){\|}^{2}}. \end{array}$$ (45)

**5**: *Testing* $F_{k-1}^{T}{\bar{y}}_{k-1}$, $\left\|F_{k-1}\right\|$ *and* $\left\|{\bar{y}}_{k-1}\right\|$. Compute $F_{k-1}^{T}{\bar{y}}_{k-1}$, $\left\|F_{k-1}\right\|$ and $\left\|{\bar{y}}_{k-1}\right\|$.

**6**: Set $d_{k}=-F_{k}+\frac{F_{k}^{T}F_{k-1}}{F_{k-1}^{T}{\bar{y}}_{k-1}}{\bar{y}}_{k-1}+\frac{F_{k}^{T}{\bar{y}}_{k-1}}{F_{k-1}^{T}{\bar{y}}_{k-1}}F_{k-1}-2\frac{\|F_{k-1}{\|}^{2}F_{k}^{T}{\bar{y}}_{k-1}}{(F_{k-1}^{T}{\bar{y}}_{k-1}{)}^{2}}{\bar{y}}_{k-1}$ if $F_{k-1}^{T}{\bar{y}}_{k-1}\geq m\|F_{k-1}\|\|{\bar{y}}_{k-1}\|$, where m∈(0,1], otherwisecompute $d_{k}=-F_{k}+\frac{F_{k}^{T}{\bar{y}}_{k-1}}{{\bar{y}}_{k-1}^{T}{\bar{y}}_{k-1}}{\bar{y}}_{k-1}-\frac{1}{r}\frac{\left|F_{k-1}^{T}{\bar{y}}_{k-1}\right|}{\left\|F_{k-1}\|\|{\bar{y}}_{k-1}\right\|}\frac{F_{k}^{T}F_{k-1}}{\|F_{k-1}{\|}^{2}}F_{k-1}$, where *r* > 1.

**7**: Set *k* = *k*+1. Goto **2**.

## 3 Analysis of convergence

We now proceed to analyze global convergence of the MKAS algorithm: First, we give the following assumptions:

**Assumption 3.1** *The mapping F is monotone.*

**Assumption 3.2** *There exists* $\bar{x}\in ℬ$ *such that* $F(\bar{x})=0$*.*

**Lemma 3.3** *The search direction* *d*_*k*_ *generated in step 6 of the MKAS algorithm satisfy the inequality*


$$\begin{array}{c} \min\left\{\left(1-\frac{1}{r}\right),\frac{1}{2}\right\}\|F_{k}\|\leq \|d_{k}\|\leq N\|F_{k}\|,\;N=\left(1+\frac{2}{m}+\frac{2}{m^{2}}\right). \end{array}$$ (46)

**Proof.** By applying (41), Cauchy-Schwarz inequality (11), the first inequality is satisfied. Now, from (40), and for *k* = 0, we have that $d_{0}=-F_{0}$, which shows that $\left\|d_{0}\|=\|F_{0}\right\|$. For *k* ≥ 1, we consider the case of $F_{k-1}^{T}{\bar{y}}_{k-1}\geq m\|F_{k-1}\|\|{\bar{y}}_{k-1}\|$. From (40) and Cauchy-Schwarz inequality, we have


$$\begin{array}{cc} \left\|d_{k}\right\| & =\|-F_{k}+\frac{F_{k}^{T}F_{k-1}}{F_{k-1}^{T}{\bar{y}}_{k-1}}{\bar{y}}_{k-1}+\frac{F_{k}^{T}{\bar{y}}_{k-1}}{F_{k-1}^{T}{\bar{y}}_{k-1}}F_{k-1}-2\frac{\|F_{k-1}{\|}^{2}F_{k}^{T}{\bar{y}}_{k-1}}{(F_{k-1}^{T}{\bar{y}}_{k-1}{)}^{2}}{\bar{y}}_{k-1}\|\\ & \leq \|F_{k}\|+\frac{\left\|F_{k}\|\|F_{k-1}\|\|{\bar{y}}_{k-1}\right\|}{F_{k-1}^{T}{\bar{y}}_{k-1}}+\frac{\left\|F_{k}\|\|F_{k-1}\|\|{\bar{y}}_{k-1}\right\|}{F_{k-1}^{T}{\bar{y}}_{k-1}}+2\frac{\|F_{k-1}{\|}^{2}\|F_{k}\|\|{\bar{y}}_{k-1}{\|}^{2}}{(F_{k-1}^{T}{\bar{y}}_{k-1}{)}^{2}}\\ & \leq \|F_{k}\|+\frac{\left\|F_{k}\|\|F_{k-1}\|\|{\bar{y}}_{k-1}\right\|}{m\|F_{k-1}\|\|{\bar{y}}_{k-1}\|}+\frac{\left\|F_{k}\|\|F_{k-1}\|\|{\bar{y}}_{k-1}\right\|}{m\|F_{k-1}\|\|{\bar{y}}_{k-1}\|}+2\frac{\|F_{k-1}{\|}^{2}\|F_{k}\|\|{\bar{y}}_{k-1}{\|}^{2}}{(m\|F_{k-1}\|\|{\bar{y}}_{k-1}\|{)}^{2}}\\ & =\|F_{k}\|+\frac{\left\|F_{k}\right\|}{m}+\frac{\left\|F_{k}\right\|}{m}+2\frac{\left\|F_{k}\right\|}{m^{2}}\\ & =\left(1+\frac{2}{m}+\frac{2}{m^{2}}\right)\|F_{k}\|. \end{array}$$

Setting $N1=\left(1+\frac{2}{m}+\frac{2}{m^{2}}\right)$, we get


$$\begin{array}{c} \|d_{k}\|\leq N1\|F_{k}\|. \end{array}$$

For the second case, we have from (40) and Cauchy Schwarz inequality, that


$$\begin{array}{cc} \left\|d_{k}\right\| & =\left\|-F_{k}+\frac{1}{r}\frac{F_{k}^{T}{\bar{y}}_{k-1}}{{\bar{y}}_{k-1}^{T}{\bar{y}}_{k-1}}{\bar{y}}_{k-1}-\frac{\left|F_{k-1}^{T}{\bar{y}}_{k-1}\right|}{\left\|F_{k-1}\|\|{\bar{y}}_{k-1}\right\|}\frac{F_{k}^{T}F_{k-1}}{\|F_{k-1}{\|}^{2}}F_{k-1}\right\|\\ & \leq \|F_{k}\|+\frac{1}{r}\frac{\|F_{k}\|\|{\bar{y}}_{k-1}{\|}^{2}}{\|{\bar{y}}_{k-1}{\|}^{2}}+\frac{\left\|F_{k-1}{\|}^{3}\|F_{k}\|\|{\bar{y}}_{k-1}\right\|}{\left\|F_{k-1}{\|}^{3}\|{\bar{y}}_{k-1}\right\|}\\ & =\left(1+\frac{1}{r}+1\right)\|F_{k}\|\\ & =\left(2+\frac{1}{r}\right)\|F_{k}\|. \end{array}$$

Setting $N2=\left(2+\frac{1}{r}\right)$, we get


$$\begin{array}{c} \|d_{k}\|\leq N2\|F_{k}\|. \end{array}$$

Therefore, since *N*1 is the max of (1,*N*1,*N*2), we obtain that


$$\begin{array}{c} \|d_{k}\|\leq \left(1+\frac{2}{m}+\frac{2}{m^{2}}\right)\|F_{k}\|, \end{array}$$ (47)

which proves that the second inequality of (46) holds.■

**Lemma 3.4** *Given that F in* (1) is c*ontinuous on* ${\mathbb{R}}^{n}$*, then for each* *k* ≥ 0*, there exists nonnegative integer* *i*_*k*_ *such that (*42) i*s satisfied.*

**Proof.** Suppose by contradiction that *k* ≥ 0 exists for which (42) does not hold for any nonnegative integer *i*, i.e.,


$$\begin{array}{cc} -F(x_{k_{0}}+\beta \rho^{i}d_{k_{0}}{)}^{T}d_{k_{0}} & <\sigma \beta \rho^{i}P_{\left[\lambda ,u\right]}\|F(x_{k_{0}}+\beta \rho^{i}d_{k_{0}})\|\|d_{k_{0}}{\|}^{2}\\ & \leq \sigma \beta \rho^{i}u\|d_{k_{0}}{\|}^{2}. \end{array}$$ (48)

The second inequality in (48) was obtained from the fact that $P_{\left[\lambda ,u\right]}[.]\leq u$. By continuity of *F*, and letting i→∞ with ρ∈(0,1), we get


$$\begin{array}{c} F(x_{k_{0}}{)}^{T}d_{k_{0}}\geq 0, \end{array}$$

which contradicts


$$\begin{array}{c} F(x_{k_{0}}{)}^{T}d_{k_{0}}\leq -\min\left\{\left(1-\frac{1}{r}\right),\frac{1}{2}\right\}\|F_{k}{\|}^{2}<0. \end{array}$$

**Lemma 3.5** *Suppose Assumptions* 3.1−3.2 *hold and the sequences* $\left\{x_{k}\right\}$ *and* $\left\{\psi_{k}\right\}$ *are generated by Algorithm 1. Then for a solution* $\bar{x}\in B$*, the sequence* $\left\{\|x_{k}-\bar{x}\|\right\}$ *is convergent and* $\left\{x_{k}\right\}$ *and* $\left\{\psi_{k}\right\}$ *are bounded.*

*Furthermore*


$$\begin{array}{c} \lim_{k\to \infty }\alpha_{k}\|d_{k}\|=0. \end{array}$$ (49)

**Proof**: Using (42) and definition of $\psi_{k}$, we have


$$\begin{array}{cc} F(\psi_{k}{)}^{T}(x_{k}-\psi_{k}) & =-\alpha_{k}F(\psi_{k}{)}^{T}d_{k}\\ & \geq \sigma \alpha_{k}^{2}P_{\left[\lambda ,u\right]}\|F(\psi_{k})\|\|d_{k}{\|}^{2}\\ & \geq \sigma \lambda \alpha_{k}^{2}\|d_{k}{\|}^{2}\\ & =\sigma \lambda \|x_{k}-\psi_{k}{\|}^{2}>0. \end{array}$$ (50)

By (2) and for all $\bar{x}\in B$, we have


$$\begin{array}{cc} F(\psi_{k}{)}^{T}(x_{k}-\bar{x}) & =F(\psi_{k}{)}^{T}(x_{k}-\psi_{k})+F(\psi_{k}{)}^{T}(\psi_{k}-\bar{x})\\ & \geq F(\psi_{k}{)}^{T}(x_{k}-\psi_{k})+F(\bar{x}{)}^{T}(\psi_{k}-\bar{x})\\ & =F(\psi_{k}{)}^{T}(x_{k}-\psi_{k})\\ & \geq \sigma \lambda \|x_{k}-\psi_{k}{\|}^{2}>0. \end{array}$$ (51)

Combining the inequality (51) above, (43), (44) and (45), we obtain


$$\begin{array}{cc} \|x_{k+1}-\bar{x}{\|}^{2} & =\|P_{ℬ}[x_{k}-\vartheta \varsigma_{k}F(\psi_{k})]-\bar{x}{\|}^{2}\\ & \leq \|x_{k}-\vartheta \varsigma_{k}F(\psi_{k})-\bar{x}{\|}^{2}\\ & =\|(x_{k}-\bar{x})-\vartheta \varsigma_{k}F(\psi_{k}){\|}^{2}\\ & =\|x_{k}-\bar{x}{\|}^{2}-2\vartheta \varsigma_{k}F(\psi_{k}{)}^{T}(x_{k}-\bar{x})+\vartheta^{2}\varsigma_{k}^{2}\|F(\psi_{k}){\|}^{2}\\ & \leq \|x_{k}-\bar{x}{\|}^{2}-2\vartheta \varsigma_{k}F(\psi_{k}{)}^{T}(x_{k}-\psi_{k})+\vartheta^{2}\varsigma_{k}^{2}\|F(\psi_{k}){\|}^{2}\\ & =\|x_{k}-\bar{x}{\|}^{2}-\vartheta (2-\vartheta )\frac{(F(\psi_{k}{)}^{T}(x_{k}-\psi_{k}){)}^{2}}{\|F(\psi_{k}){\|}^{2}}\\ & \leq \|x_{k}-\bar{x}{\|}^{2}-\vartheta (2-\vartheta )\frac{\sigma^{2}\lambda^{2}\|x_{k}-\psi_{k}{\|}^{4}}{\|F(\psi_{k}){\|}^{2}}, \end{array}$$ (52)

where the last inequality indicates that $0\leq \|x_{k+1}-\bar{x}\|\leq \|x_{k}-\bar{x}\|$ holds. The implication of this is that $\left\{\|x_{k}-\bar{x}\|\right\}$ is a monotone decreasing sequence that is bounded below. So, $\left\{\|x_{k}-\bar{x}\|\right\}$ is convergent and $\left\{x_{k}\right\}$ is bounded. Now, since $\left\{x_{k}\right\}$ is bounded and *F* is continuous, we see that there exists a constant $\bar{\vartheta }>0$ such that for all *k* ≥ 0,


$$\begin{array}{c} \|x_{k}\|\leq \bar{\vartheta },\;\|F(x_{k})\|\leq \bar{\vartheta }. \end{array}$$ (53)

Combining (53) with (47) shows that $\left\{d_{k}\right\}$ is bounded. Furthermore, from (2), Cauchy Schwarz inequality, and (50), we have


$$\begin{array}{cc} \bar{\vartheta } & \geq \|F_{k}\|\geq \frac{F_{k}^{T}(x_{k}-\psi_{k})}{\left\|x_{k}-\psi_{k}\right\|}\geq \frac{F(\psi_{k}{)}^{T}(x_{k}-\psi_{k})}{\left\|x_{k}-\psi_{k}\right\|}\geq \sigma \lambda \|x_{k}-\psi_{k}\|\\ & \geq \sigma \lambda \|\psi_{k}\|-\sigma \lambda \bar{\vartheta }, \end{array}$$

which further yields


$$\begin{array}{c} \|\psi_{k}\|\leq \frac{\bar{\vartheta }+\sigma \lambda \bar{\vartheta }}{\sigma \lambda }, \end{array}$$

which clearly shows the boundedness of $\left\{\psi_{k}\right\}$, which by continuity of *F*, indicates boundedness of $\left\{F(\psi_{k})\right\}$, namely, a constant κ exists for which


$$\begin{array}{c} \|F(\psi_{k})\|\leq \kappa .\;\forall k\geq 0. \end{array}$$

Combining this result with the last inequality of (52) yields


$$\begin{array}{cc} \vartheta (2-\vartheta )\frac{\sigma^{2}\lambda^{2}}{\kappa^{2}}\sum_{k=0}^{\infty }\|x_{k}-\psi_{k}{\|}^{4} & \leq \sum_{k=0}^{\infty }(\|x_{k}-\bar{x}{\|}^{2}-\|x_{k+1}-\bar{x}{\|}^{2})\\ & =\|x_{0}-\bar{x}{\|}^{2}-\lim_{k\;\to \;\infty }\|x_{k+1}-\bar{x}{\|}^{2}<\infty , \end{array}$$

which further implies that $0\leq \lim_{k\;\to \;\infty }\|x_{k}-\psi_{k}\|=\lim_{k\;\to \;\infty }\alpha_{k}\|d_{k}\|$. Hence the proof is obtained.■

**Theorem 3.6** *The sequence* $\left\{x_{k}\right\}$ *generated by MKAS Algorithm converges globally to a solution of (1).*

**Proof.** Two cases are analyzed for the proof

1^*st*^ case: Assuming that ${lim inf}_{k\to 0}\|F_{k}\|=0$, then the boundedness of $\left\{x_{k}\right\}$ and continuity of *F* implies that some cluster points $\hat{x}$ exists for which $F(\hat{x})=0$. Also, since $\left\{\|x_{k}-\hat{x}\|\right\}$ is convergent, it implies that $\left\{x_{k}\right\}$ converges to $\hat{x}$.

2^*nd*^ case: ${lim inf}_{k\to 0}\|F_{k}\|>0$. In this case we have that a constant $\bar{c}>0$ exists such that


$$\begin{array}{c} \|F_{k}\|\geq \bar{c},\;\forall k\geq 0. \end{array}$$ (54)

From first inequality in (46), we have


$$\begin{array}{c} \|d_{k}\|\geq \min\left\{\left(1-\frac{1}{r}\right),\frac{1}{2}\right\}\|F_{k}\|\geq \min\left\{\left(1-\frac{1}{r}\right),\frac{1}{2}\right\}\bar{c}. \end{array}$$

Combining this result with (49) yields


$$\begin{array}{c} \lim_{k\;\to \;\infty }\alpha_{k}=0. \end{array}$$ (55)

By the boundedness of the sequences $\left\{x_{k}\right\}$ and $\left\{d_{k}\right\}$, we see that there exists subsequences $\left\{x_{k_{i}}\right\}$ and $\left\{d_{k_{i}}\right\}$ for which


$$\begin{array}{c} \lim_{i\;\to \;\infty ,i\in 𝒦}x_{k_{i}}=\hat{x},\;\lim_{i\;\to \;\infty ,i\in 𝒦}d_{k_{i}}=\hat{d}, \end{array}$$

with 𝒦 being an infinite indexing set. From (46), we get


$$\begin{array}{c} -F(x_{k_{i}}{)}^{T}d_{k_{i}}\geq \min\left\{\left(1-\frac{1}{r}\right),\frac{1}{2}\right\}\|F(x_{k_{i}}){\|}^{2},\;\forall i\in 𝒦. \end{array}$$ (56)

Letting i→∞ in (56) with continuity of *F* and (54), we obtain


$$\begin{array}{c} -F(\hat{x}{)}^{T}\hat{d}\geq \min\left\{\left(1-\frac{1}{r}\right),\frac{1}{2}\right\}\|F(\hat{x}){\|}^{2}>\min\left\{\left(1-\frac{1}{r}\right),\frac{1}{2}\right\}{\bar{c}}^{2}>0. \end{array}$$ (57)

On the other hand, from (42), if $\alpha_{k}\neq \beta$, then $\bar{\alpha }=\frac{\alpha }{\rho }$ will not satisfy (42), i.e.,


$$\begin{array}{cc} -F(x_{k_{i}}+\rho^{-1}\alpha_{k_{i}}d_{k_{i}}{)}^{T}d_{k_{i}} & <\sigma \rho^{-1}\alpha_{k_{i}}P_{\left[\lambda ,u\right]}[\|F(x_{k_{i}}+\rho^{-1}\alpha_{k_{i}}d_{k_{i}})\|]\|d_{k_{i}}{\|}^{2}\\ & \leq \sigma \rho^{-1}\alpha_{k_{i}}u\|d_{k_{i}}{\|}^{2} \end{array}$$ (58)

By taking limit as i→∞, with i∈𝒦 in (58), (55) and continuity of *F*, we obtain


$$\begin{array}{c} -F(\hat{x}{)}^{T}\hat{d}\leq 0, \end{array}$$

which shows a contradiction with (57). Hence the proof is established.■

## 4 Numerical experiments

To test the MKAS method’s effectiveness, it is compared with the QN-type methods in [55] labelled SMDFP, [57] labelled SMBFGS and [56] labelled PBDFP. To code the four algorithms, MATLAB *R*2014*a* was employed with a PC configured as (2.30ghz cpu, 4GB RAM). The criteria for stopping the iterations is $\|F(x_{k})\|\leq 10^{-10}$ or $\|F(\psi_{k})\|\leq 10^{-10}$ or iterations exceed 1000 without attaining a solution. Parameters values for MKAS are set as β=1, ρ=0.5, σ=0.01, ϑ=1.99, ν=0.001, *m* = 0.01, *r* = 1.6, λ=0.001 and *u* = 0.8. Parameter values for the other algorithms are set according to how they are used in the papers.

The following test examples with dimensions 1000, 5000, and 10000, where *F* is given as: $F=(f_{1}(x),f_{2}(x),...,f_{n}(x){)}^{T}$.

**Example 1** This is the gradient of the strictly convex test example in [70] where $ℬ={\mathbb{R}}_{+}^{n}$ added to yield

$f_{i}(x)=\exp x_{i}-1$, i=1,2,…,n.

**Example 2** This is a modified version of the test example in [71] with $ℬ={\mathbb{R}}_{+}^{n}$ added to yield

$f_{1}(x)=\exp x_{1}-1$,

$f_{i}(x)=\exp x_{i}+x_{i}-1$, i=2,…,n.

**Example 3** [72] with $ℬ={\mathbb{R}}_{+}^{n}$

$f_{i}(x)=2x_{i}-\sin\left|x_{i}\right|$, i=1,2,…,n.

**Example 4** [73].

$f_{1}(x)=x_{1}-\exp\left(cos\left(\frac{x_{1}+x_{2}}{2}\right)\right)$,

$f_{i}(x)=x_{i}-\exp\left(cos\left(\frac{x_{i-1}+x_{i}+x_{i+1}}{i}\right)\right),\;i=2,3,\ldots ,n-1$,

$f_{n}(x)=x_{n}-\exp\left(cos\left(\frac{x_{n-1}+x_{n}}{n}\right)\right)$,

with $ℬ={\mathbb{R}}_{+}^{n}$.

**Example 5** A modified version of the test example in [72] with $ℬ={\mathbb{R}}_{+}^{n}$

$f_{i}(x)=3x_{i}-\sin x_{i}$, i=1,2,…,n.

**Example 6** A modification of the test example 3 with $ℬ={\mathbb{R}}_{+}^{n}$

$f_{i}(x)=3x_{i}+\cos x_{i}-1$, i=1,2,…,n.

**Example 7** A modification of the test example 3 with $ℬ={\mathbb{R}}_{+}^{n}$ added to yield

$f_{1}(x)=2x_{1}+\exp\sin x_{1}-1$,

$f_{i}(x)=2x_{i-1}+\exp\sin x_{i}+2x_{i}-1$,

$f_{n}(x)=2x_{n}+\exp\sin x_{n}-1$,i=2,…,n−1.

**Example 8** A modification of the test example 3 with $ℬ={\mathbb{R}}_{+}^{n}$ added to yield

$f_{1}(x)=\sin x_{1}+\exp\sin x_{1}-1$,

$f_{i}(x)=\sin x_{i}+\exp\sin x_{i}+x_{i}-1$, i=2,…,n.

**Example 9** A modification of the test example 2 with $ℬ={\mathbb{R}}_{+}^{n}$ added to yield

$f_{1}(x)=3x_{1}+\exp\sin x_{1}-1$,

$f_{i}(x)=3x_{i}+\exp\sin x_{i}-1$,i=2,…,n.

The following initial guesses were used:

***x***_**1**_$={\left(\frac{1}{n},\frac{2}{n},...,1\right)}^{T}$, ***x***_**2**_$={\left(1,\frac{1}{2},...,\frac{1}{n}\right)}^{T}$, ***x***_**3**_ $={\left(\frac{1}{2},\frac{3}{2},...,-\frac{\left[(-1{)}^{n}-2\right]}{2}\right)}^{T}$, ***x***_**4**_ $=(1,1,...,...,1{)}^{T}$,

***x***_**5**_ $={\left(\frac{3}{4},\frac{1}{4},...,-\frac{\left[(-1{)}^{n}-2\right]}{4}\right)}^{T}$, ***x***_**6**_ $=(2,2,...,2{)}^{T}$. The numerical result of the experiments for the four algorithms is displayed in three tables, and is accessible from the link https://github.com/hungugida/hungugida/blob/main/Tables1-3MKAS.pdf, where “N” and “Pdim” denotes example of the operator *F* and dimension, “SP” and “Nit” represents for initial guess and number of iterations. Also, “Fval,” “Ptime(s)” and “Norm” stands for function evaluations, processing time and the residual at stopping point, while “***” denotes failure to obtain a solution.

**Fig 1:**
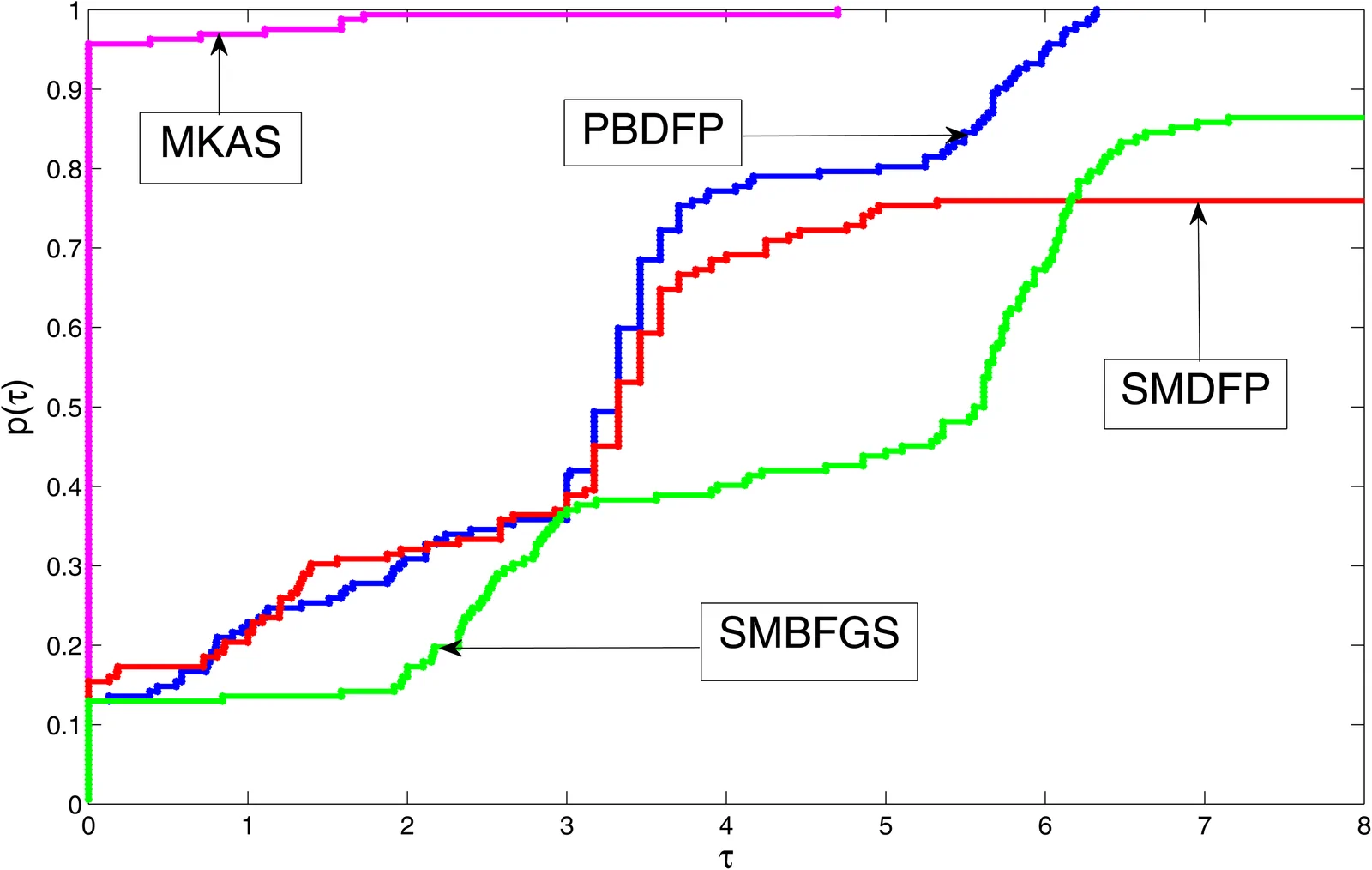
Performance profile of the four methods for number of iterations.

**Fig 2:**
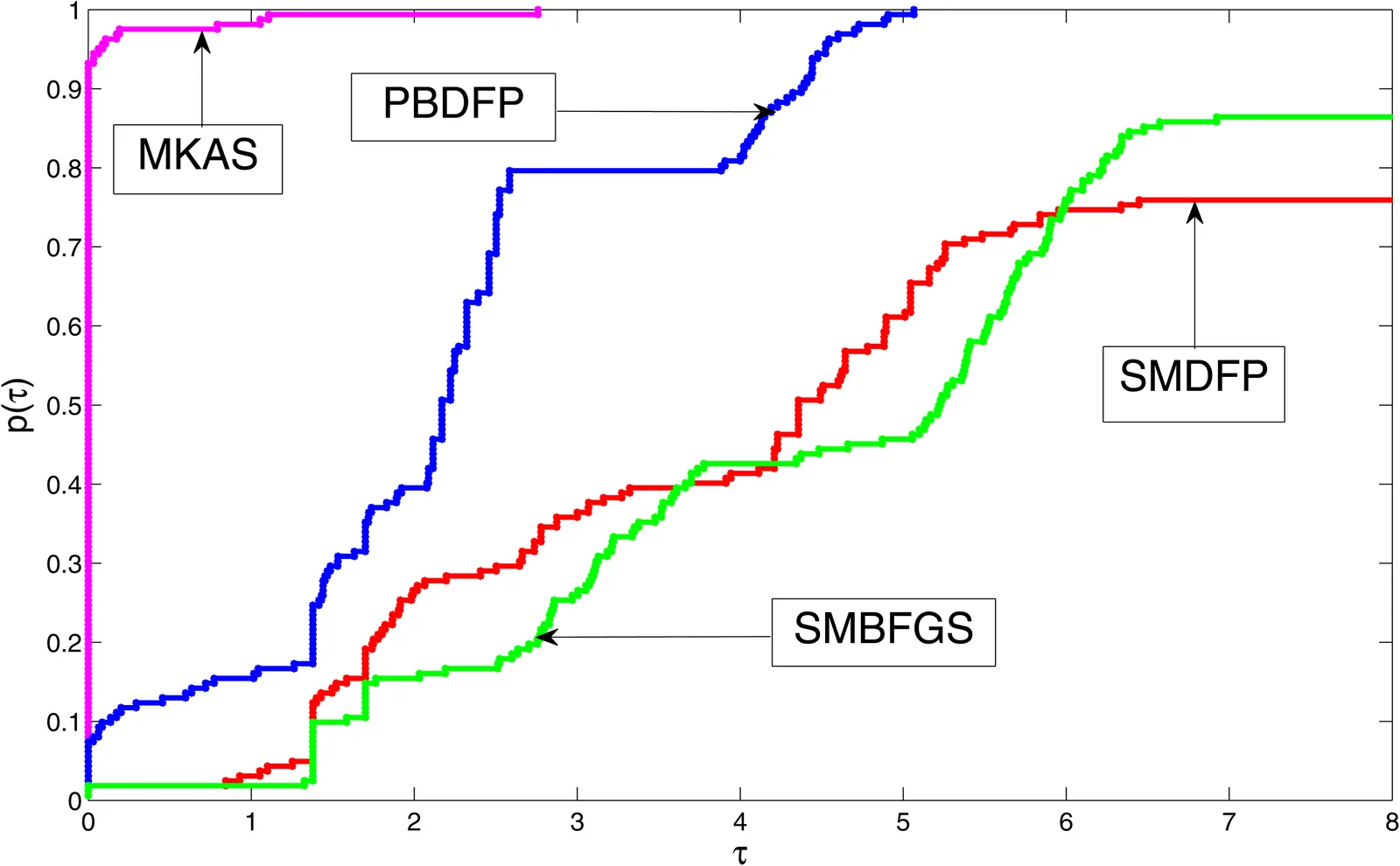
Performance profile of the four methods for function evaluations.

**Fig 3:**
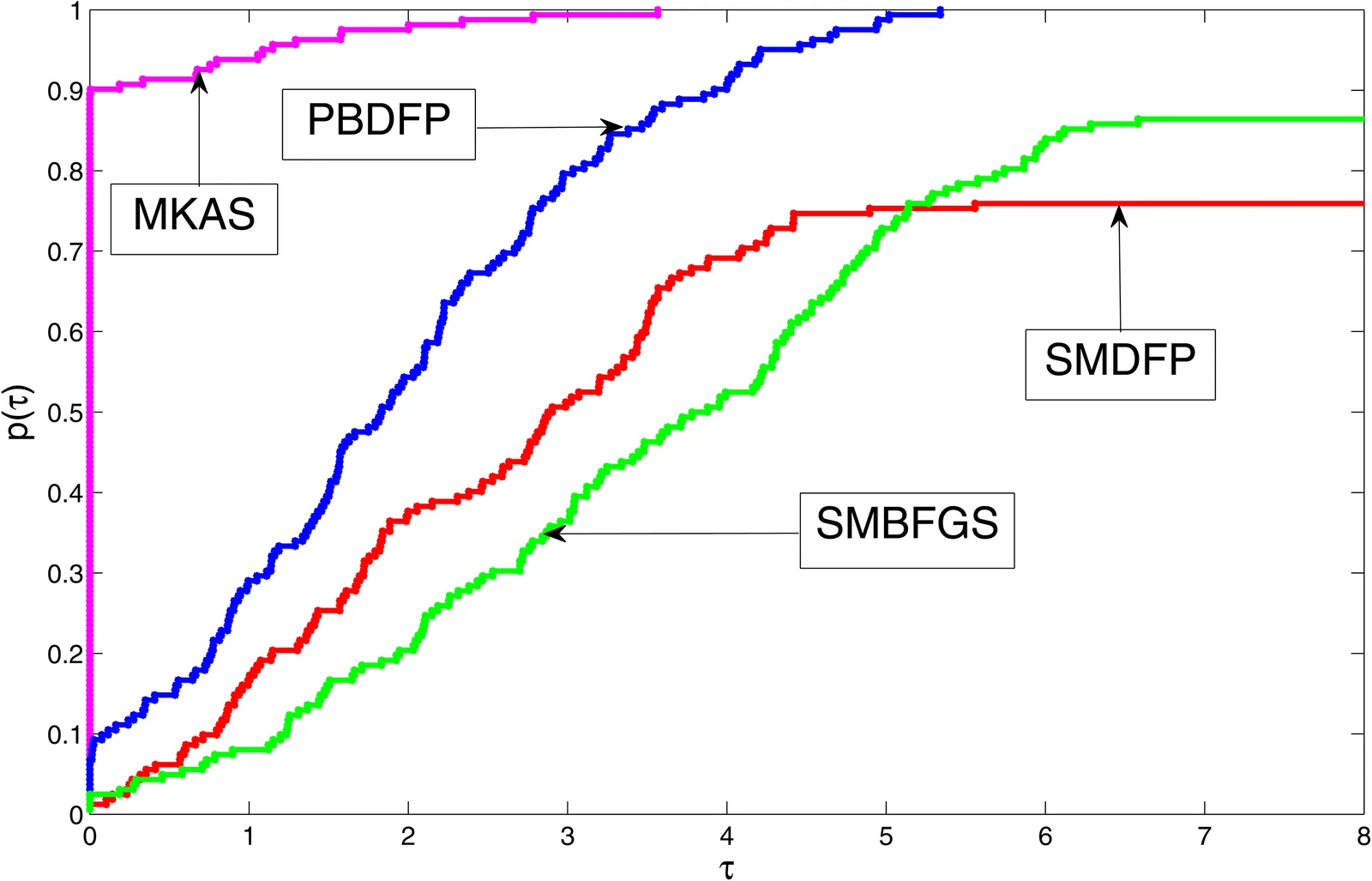
Performance profile of the four methods for CPU time.

In order describe the values shown in the above link, we use the evaluation too by Dolan and Moré [74]. Figs 1–3 were plotted for iterations, function evaluations, and CPU time performance metrics. From Fig 1, we see that MKAS solved about 95% of the test examples with less number of iterations. This values however, include 9% solved by the algorithm together with PBDFP, SMDFP, and SMBFGS. It also showed that SMDFP solved 15% of the test examples with less iterations. Here also, this value includes 12% solved with the other algorithms. Fig 2 showed that MKAS solved 93% of the test examples with less function evaluations. It also showed that PBDFP solved 8%, which includes 2% solved with SMDFP, and SMBFGS algorithms. For least CPU time metric, Fig 3 showed that MKAS perform better than PBDFP, SMDFP, and SMBFGS because it solved 89% while the latter recorded 8%, 1% and 2%. To further validate the results of the experiments, we present the average residual obtained for each of the four algorithms as computed from tables in the above link as follows: MKAS (1.21 × 10^−11^), PBDFP (4.41 × 10^−11^), SMDFP (1.83 × 10^−10^) and SMBFGS (1.75 × 10^−10^). Hence, the above analysis indicates that MKAS method is better suited for solving (1) than the other schemes.

## 5 Concluding remarks and future research

We have presented a new quasi-Newton-type method for solving constrained system of monotone nonlinear equations in this article. The method is an adaptive modification of the BFGS method for unconstrained optimization. The scheme possesses the vital property for analyzing global convergence as well as the trust region property irrespective of the line search procedure; what’s more, the global convergence was proven without the Lipschitz condition, thereby broadening its applicability to a wider class of problems. Another important finding was the impact of the restart strategy in improving convergence of the scheme, as evident in the best average residual it recorded. Furthermore, numerical experiments with the method and some of the available quasi-Newton type methods for solving constrained system of monotone nonlinear equations showed that it is effective. The focus of a future research will be to develop a variant of the proposed method, where the global convergence will be analyzed without the monotonicity and Lipschitz assumptions. We also hope to include real-life applications of the method in the study.
