# Correction: A novel adaptive quasi-Newton-type update and its global convergence without Lipschitz condition for constrained system of nonlinear monotone equations

**DOI:** 10.1371/journal.pone.0362707

**Published:** 2026-11-10

**Authors:** The PLOS One Staff

## Notice of Republication

This article was republished on October 22^nd^, 2026, to correct errors in author affiliations, equation references and the financial disclosure.

The publisher apologizes for the errors. Please download this article again to view the correct version. The originally published, uncorrected article and the republished, corrected articles are provided here for reference.

## Supporting information

S1 File Originally published, uncorrected article. (PDF)

S2 File Republished, corrected article. (PDF)
