# Supplementary material for: Correction: A novel adaptive quasi-Newton-type update and its global convergence without Lipschitz condition for constrained system of nonlinear monotone equations
Source: PLoS One. 2026 Nov 10;21(11):e0362707. doi: 10.1371/journal.pone.0362707 (PMC13750584; doi:10.1371/journal.pone.0362707)
Supplement: Supplementary file 2 [file pone.0362707.s002.pdf]

PLOS One

RESEARCH ARTICLE

A novel adaptive quasi-Newton-type update and its global convergence without Lipschitz condition for constrained system of nonlinear monotone equations

Kabiru Ahmed[1,2], Hatem E. Semary[3], Asmaa S. Al-Moisheer[3], Sulaiman M. Ibrahim[4,5]*, Abubakar Sani Halilu[6], Muhammed Yusuf Waziri[1], Mohamad Afendee Mohamed[6], Salisu Murtala[2]

1 Department of Mathematical Sciences, Bayero University, Kano, Nigeria, **2** Department of Mathematical Sciences, Federal University, Dutse, Nigeria, **3** Department of Mathematics and Statistics, Faculty of Science, Imam Mohammad Ibn Saud Islamic University (IMSIU), Riyadh, Saudi Arabia, **4** College of Applied and Health Sciences, A'Sharqiyah University, Ibra, Sultanate of Oman, **5** Faculty of Education and Arts, Sohar University, Sohar, Oman, **6** Faculty of Informatics and Computing, Universiti Sultan Zainal Abidin, Terengganu, Malaysia

* sulaimancga@gmail.com

Check for updates

OPEN ACCESS

Citation: Ahmed K, Semary HE, Al-Moisheer AS, Ibrahim SM, Halilu AS, Waziri MY, et al. (2026) A novel adaptive quasi-Newton-type update and its global convergence without Lipschitz condition for constrained system of nonlinear monotone equations. PLoS One 21(6): e0344697. https://doi.org/10.1371/journal.pone.0344697

Editor: Md. Kamrujjaman, University of Dhaka, BANGLADESH

Received: October 7, 2025

Accepted: February 24, 2026

Published: June 10, 2026

Copyright: © 2026 Ahmed et al. This is an open access article distributed under the terms of the Creative Commons Attribution License, which permits unrestricted use, distribution, and reproduction in any medium, provided the original author and source are credited.

Data availability statement: All relevant data generated and analyzed can be assessed using the following link https://github.com/hungugida/hungugida/blob/main/Tables1-3 MKAS.pdf.

Abstract

This paper presents a new iterative method with a restart feature for solving constrained system of nonlinear monotone equations. The scheme, which is a double-parameter method, was initiated by considering a positive-definite adaptation of the quasi-Newton update proposed by Andrei (J. Comput. Appl. Math. 332, 26–44 (2018)) for unconstrained optimization. The two scaling parameters of the method are obtained by employing the measure function by Byrd and Nocedal (SIAM J. Numer. Anal. 26, 727–739 (1989)), which ensures that the condition number of the update is minimized. Another important attribute of the method is that its global convergence analysis is conducted without the Lipschitz assumption, which is a strong condition. Furthermore, the two parameters embedded in the scheme help in maintaining a balance in the distribution of the eigenvalues of its update matrix. The method converges globally regardless of the line search procedure employed. Numerical experiments with some methods in the literature show that the scheme is effective.

1 Introduction

The interest in this paper is solution of the constrained system of nonlinear equations

$$F(\bar{x}) = 0, \quad \bar{x} \in \mathcal{B} \subseteq \mathbb{R}^n, \tag{1}$$

where $\mathcal{B}$ is a nonempty closed convex set and F is a continuous vector-valued mapping, which is also monotone, namely, it satisfies

Funding: This work was supported and funded by the Deanship of Scientific Research at Imam Mohammad Ibn Saud Islamic University (IMSIU) (grant number IMSIU-DDRSP2601).

Competing interests: The authors have declared that no competing interests exist.

$$(F(x) - F(y))^T(x - y) \geq 0, \quad \forall x, y \in \mathbb{R}^n. \tag{2}$$

Real life applications of (1) can be found in studies such as general equilibrium problems in [1,2], the control systems analyzed in [3], networking and communications [4], modelling and data estimation [5], and in compressed sensing problems [6–14]. Some of the methods employed in solving (1) include adaptive versions of Newton's method and its enhanced version, the quasi-Newton (QN) schemes [15–18], the derivative-free methods [19–24], Levenberg-Marquardt method [25], trust-region method [26] as well as spectral projection methods [27,28]. Despite their fast convergence properties, adaptations of Newton and QN methods have not been widely studied and few of the schemes for solving (1) have been developed. A viable reason for this is the huge memory required at each iteration to store matrices generated by the methods. The classical Newton and QN methods were developed to solve the optimization problem

$$\min_{x \in \mathbb{R}^n} f(x), \tag{3}$$

with f being a smooth real-valued nonlinear function, with gradient $g(x) = \nabla f(x)$. The sequence of iterates generated by these methods are obtained via the formula

$$x_{k+1} = x_k + \alpha_k d_k, \quad k = 0, 1, 2 \ldots,$$

where α_k represents a step-size that is usually calculated using a line search procedure, and $d_k \in R^n$ is search direction that is computed via

$$G_k d_k = -\nabla f(x_k),$$

in Newton's method, or

$$B_k d_k = -\nabla f(x_k), \tag{4}$$

in QN method, where $G_k \in R^{n\times n} = \nabla^2 f(x_k)$ is a matrix known as the true Hessian or its approximation B_k in the QN method. The first QN update was developed by Davidon [29] with subsequent analysis by Fletcher and Powell [30], hence the name DFP after the three authors. The update is defined by

$$H_{k+1} = H_k - \frac{H_k y_k y_k^T H_k}{y_k^T H_k y_k} + \frac{s_k s_k^T}{y_k^T s_k}, \tag{5}$$

in which $y_k = g_{k+1} - g_k$, $s_k = x_{k+1} - x_k$. By setting H_k to be the identity matrix, the memoryless version of (5) is obtained, namely

$$H_{k+1} = I_k - \frac{y_k y_k^T}{y_k^T y_k} + \frac{s_k s_k^T}{y_k^T s_k}.$$

A DFP scheme with the update (5) exhibit some nice properties, which includes quadratic termination, hereditary property, and generating conjugate directions for a quadratic objective function and an exact line search procedure. It retains positive-definiteness for general nonlinear functions, when the curvature condition $s_k^T y_k > 0$ is satisfied. Iterations of the scheme require $3n^2 + \mathcal{O}(n)$ and it has super-linear rate of convergence. Also, for strictly convex functions under the exact line search rule, the DFP scheme converges globally [31]. Notwithstanding these attributes, numerical studies and computations conducted have shown that the DFP update is usually unstable. This is due to the loss of positive-definiteness along some iterative points as well as the distribution of its eigenvalues. The Broyden [32], Fletcher [33], Goldfarb [34], and Shanno [35] (BFGS) update is the most prominent QN scheme available. Being a member of the Broyden family, the BFGS update is given by

$$B_{k+1} = B_k - \frac{B_k s_k s_k^T B_k}{s_k^T B_k s_k} + \frac{y_k y_k^T}{y_k^T s_k}, \quad k = 0, 1, 2 \ldots, \tag{6}$$

where s_k and y_k remain as defined in (5). For the purpose of practical computations, search directions of the QN methods (4) are usually expressed in closed form as

$$d_{k+1} = -H_{k+1} g_{k+1}, \tag{7}$$

where $H_{k+1} = B_{k+1}^{-1}$ denotes the inverse approximation to the Hessian of the BFGS method. By applying the popular Woodbury formula [36] twice on (6), the inverse is obtained as

$$H_{k+1} = H_k - \frac{s_k y_k^T H_k + H_k y_k s_k^T}{s_k^T y_k} + \left(1 + \frac{y_k^T H_k y_k}{s_k^T y_k}\right) \frac{s_k s_k^T}{s_k^T y_k}. \tag{8}$$

In order to avoid computing the matrix H_k, which involves huge storage at each iteration, it is replaced with the identity matrix I and the so called memoryless update is obtained, i.e.,

$$H_{k+1} = I - \frac{s_k y_k^T + y_k s_k^T}{s_k^T y_k} + \left(1 + \frac{\|y_k\|^2}{s_k^T y_k}\right) \frac{s_k s_k^T}{s_k^T y_k}. \tag{9}$$

Further analysis of (6) by Andrei [37] shows that efficiency of the BFGS method depends so much on structure of its eigenvalues. It was also shown by Powell [38] and Byrd et al. [39] that the BFGS method suffers more from large eigenvalues of (6) than small ones. By conducting some numerical experiments on the BFGS method, Gill and Leonard [40] discovered that large number of iterations or functions and gradient evaluations may be required on some problems for the scheme to converge. These shortcomings of the scheme were shown in [40] to mostly emanate from poor initial Hessian approximations or ill-conditioning along the iterations. To address these problems, some scaling procedures were applied on the update in (6). By scaling the third term in (6) with a positive parameter γ_k, Biggs [41] gave the modification

$$B_{k+1} = B_k - \frac{B_k s_k s_k^T B_k}{s_k^T B_k s_k} + \gamma_k \frac{y_k y_k^T}{y_k^T s_k}.$$

Oren and Luenberger [42] scaled the first two terms of the update in (6) to obtain

$$B_{k+1} = \delta_k \left[B_k - \frac{B_k s_k s_k^T B_k}{s_k^T B_k s_k} \right] + \frac{y_k y_k^T}{y_k^T s_k},$$

where δ_k is a positive parameter. By adopting the technique of changing structure of eigenvalues Andrei [43] presented a two-parameter BFGS method, with B_{k+1} given as

$$B_{k+1} = \delta_k \left[B_k - \frac{B_k s_k s_k^T B_k}{s_k^T B_k s_k} \right] + \gamma_k \frac{y_k y_k^T}{y_k^T s_k},$$

with δ_k and γ_k being positive parameters. Here, δ_k is computed to ensure that eigenvalues of B_{k+1} are clustered, while γ_k is obtained so that large eigenvalues of B_{k+1} are reduced, i.e., the eigenvalues are shifted to the left. This strategy brings about a better distribution of the eigenvalues. A modification of (9) was proposed in [44], namely

$$H_{k+1} = \frac{1}{\delta_k} \left[H_k - \frac{H_k y_k s_k^T + s_k y_k^T H_k}{s_k^T y_k} + \left(\frac{\delta_k}{\gamma_k} + \frac{y_k^T H_k y_k}{s_k^T y_k} \right) \frac{s_k s_k^T}{s_k^T y_k} \right], \tag{10}$$

where δ_k and γ_k are parameters that are determined by employing Byrd and Nocedal's measure function in [45]. The method proves to be computationally cheap and also has superlinear rate of convergence on practical problems.

In order to address some of the shortfalls of the DFP (5) and BFGS (6) updates and address some of their shortfalls, some variants for solving problem (3) have been proposed in the literature. Based on the ℓ_∞ matrix norm and an upper bound for condition number of the scaled version of (9), Babaie-Kafaki [46] proposed a BFGS-type method for the unconstrained optimization problem (3). The author proved that the method satisfy the descent condition and conducted numerical experiments to show its effectiveness. Also, Babaie-Kafaki and Aminifard [47] proposed two parameter scaled memoryless BFGS update in (8) for the problem (3) with a monotone choice for the initial step length. The authors proved the sufficient descent condition of the scheme for uniformly convex functions. Aminifard and Babaie-Kafaki [48] developed a DFP-type method for solving (3) by analyzing eigenvalues of a scaled memoryless variant of the DFP update (5), where the scaled parameter is obtained by skipping the direction of maximum magnification. By exploiting a spectral secant condition for the classical DFP update (5), Dargahi et al. [49] proposed a DFP-type method for (3), where the updating matrix is obtained such that its condition number is minimized. Bakhshinejad and Babaie-Kafaki [50] also proposed a DFP-type algorithm as an extension of the search direction obtained by applying the DFP update (5). The method was shown to satisfy the much required sufficient descent condition.

In order to exploit some of the advantages of the DFP and BFGS updates and address their shortcomings, some adaptive versions for solving (1) and its unconstrained form have been proposed in recent years. In line with this, Mamat et al. [51] proposed a DFP-type algorithm for solving the symmetric form of problem (1) by employing a scaled memoryless version of the DFP update (5). The authors proved global convergence of the scheme by employing some basic assumptions. As an enhancement of the classical DFP update (5), Dauda [52] proposed a three-term DFP-type method for solving the symmetric form of (1). The authors proved its global convergence using some mild conditions. Based on a scaled memoryless version of the DFP update (5), Awwal et al. [53] proposed a derivative-free method for solving (1). The authors proved global convergence of the method and compared its performance with some methods for solving (1). Inspired by a scaled version of the memoryless DFP update (5), another derivative-free method was proposed by Rao and Huang [54] for the unconstrained form of (1). Global convergence of the method was shown by employing some mild conditions. By applying a scaling technique and the measure function of Byrd and Nocedal [45] on a scaled memoryless DFP update (5), Ullah et al. [55] proposed a derivative-free DFP-type method for the constrained problem (1). Both global convergence and numerical efficiency were shown by the authors. In a

recent development, Rehman et al. [56] proposed another version of a derivative-free DFP method for solving (1). The method converges globally and its effectiveness was illustrated by comparison with some recent methods in the literature. Recently, by computing an optimal value for the scaled memoryless BFGS update (9), Shah et al. [57] proposed a scaled derivative-free BFGS method for solving (1). The authors proved global convergence of the scheme and demonstrated its application to motion control. For more recent methods for solving (1), the reader can refer to works in [58–65]. The objectives of this paper are as follows:

- To develop a QN-type algorithm close to the BFGS method, for solving problem (1), which avoids the explicit computation of any matrix in the algorithm.
- To develop a scheme that exhibits some nice attributes of the classical BFGS update, addresses some of its shortcomings, and which possess the vital property for analyzing the convergence for problem (1).
- To develop a globally convergent algorithm without the Lipschitz condition and to analyze its numerical performance in solving problem (1).

The paper is structured as follows: Motivation and details of the method are given in the following section. Analysis of its global convergence is given in Section 3. Results of some numerical experiments of the scheme and some recent methods in the literature are presented in Section 4. Conclusions are given in Section 5.

2 An adaptive QN-type method

An adaptive QN-type scheme will be derived in this section. To achieve that, the following lemma is imperative:

Lemma 2.1. ***Cauchy Schwarz inequality***
Let $\overline{V}$ be an inner product space over the field of complex numbers $\mathbb{C}$ with inner product $\langle .,. \rangle$. Then, for every pair of vectors $x, y \in \overline{V}$, the following inequality holds.

$$|\langle x, y\rangle|^2 \leq \langle x, x\rangle\langle y, y\rangle. \tag{11}$$

As stated in the introduction section, adaptations of the iterative methods for the problem in (3) have been developed over the years for solving (1). However, to the best of our knowledge few QN-type schemes exists in the literature. The ones available include the derivative-free DFP-type method proposed by Awwal et al. [53], where the authors scaled the first term of the classical DFP update (5) with a positive parameter γ_k to present

$$H_{k+1} = \mu_k H_k - \frac{H_k y_k y_k^T H_k}{y_k^T H_k y_k} + \frac{s_k s_k^T}{y_k^T s_k}, \quad k = 0, 1, 2 \ldots, \tag{12}$$

where μ_k represents a positive parameter that is computed such that the condition for analyzing global convergence holds for the scheme. Setting $H_k = \gamma_k I$ in (12) yields

$$H_{k+1} = \mu_k \gamma_k I - \frac{\gamma_k y_k y_k^T}{y_k^T y_k} + \frac{s_k s_k^T}{y_k^T s_k}, \quad k = 0, 1, 2 \ldots, \tag{13}$$

where $\gamma_k \in [\bar{\gamma}_1, \bar{\gamma}_2]$, $\bar{\gamma}_1, \bar{\gamma}_2 > 0$ is a scaling parameter. By substituting (13) into (7), the authors obtained the search direction

$$d_{k+1} = -\mu_k \gamma_k F_{k+1} - \frac{s_k^T F_{k+1}}{s_k^T y_k} s_k + \gamma_k \frac{y_k^T F_{k+1}}{y_k^T y_k} y_k, \quad k = 0, 1, 2 \ldots,$$

with $\mu_k \geq \alpha + 1$, $\alpha > 0$. The choice of μ_k suggested by the authors only guarantees the condition for analyzing global convergence holds, it does not ensure stability of the proposed method. Following the work by Awwal et al. [53], Ullah et al. [55] scaled the third term of the memoryless version of the DFP update (5) with a positive parameter γ_k to obtain

$$H_{k+1} = I_n - \frac{y_k y_k^T}{y_k^T y_k} + \gamma_k \frac{s_k s_k^T}{y_k^T s_k}, \quad k = 0, 1, 2\dots, \tag{14}$$

where γ_k,which is derived by employing the measure function of Nocedal and Byrd [45] is obtained as $\frac{s_k^T y_k}{\|s_k\|^2}$. Using this value of γ_k, the authors presented search direction of the scheme as

$$d_{k+1} = -F_{k+1} + \frac{F_{k+1}^T y_k}{\|y_k\|^2} y_k - \frac{F_{k+1}^T s_k}{\|s_k\|^2} s_k. \tag{15}$$

The value of the scaling parameter γ_k obtained reduces the chances of the DFP updating matrix from being ill-conditioned. Still, it does not ensure that the search direction (15) satisfies the condition for analyzing global convergence. Following this, Shah et. al. [57] proposed another QN-type method for solving (1) by scaling the first and second term of the memoryless version of the Hessian approximation of the BFGS update [66], namely

$$B_{k+1} = \gamma_k I - \gamma_k \frac{s_k s_k^T}{s_k^T s_k} + \frac{y_k y_k^T}{y_k^T s_k}, \quad k = 0, 1, 2\dots. \tag{16}$$

The authors minimized the condition number of (16) to obtain the choice of γ_k as $\frac{\|y_k\|}{\|s_k\|}$ resulting in the search direction

$$d_{k+1} = -\frac{\|s_k\|}{\|y_k\|} F_{k+1} + \left(\frac{s_k^T F_{k+1} y_k + y_k^T F_{k+1} s_k}{y_k^T s_k} \right) \frac{\|s_k\|}{\|y_k\|} - \frac{\|y_k\|^2 s_k^T F_{k+1} s_k}{(y_k^T s_k)^2} \frac{\|s_k\|}{\|y_k\|} - \frac{s_k^T F_{k+1} s_k}{y_k^T s_k}.$$

In (16), the parameter γ_k does not always ensure stability of the BFGS method as the third term on the right may grow larger producing a shift of the eigenvalues to the right. In a recent attempt to address the shortfalls of the DFP update (5), Rehman et. al. [56] scaled the first and second terms of the memoryless DFP update (5), namely

$$H_{k+1} = \gamma_k I_n - \gamma_k \frac{y_k y_k^T}{y_k^T y_k} + \frac{s_k s_k^T}{y_k^T s_k}, \quad k = 0, 1, 2\dots, \tag{17}$$

with γ_k being a positive parameter whose optimal value was obtained as the minimizer of the condition number of H_{k+1} which is given as

$$\gamma_k = \frac{\|s_k\|}{\|y_k\|}. \tag{18}$$

By substituting (18) into the memoryless DFP update (17), the authors obtained

$$H_{k+1} = \frac{\|s_k\|}{\|y_k\|} I_n - \frac{\|s_k\|}{\|y_k\|} \frac{y_k y_k^T}{y_k^T y_k} + \frac{s_k s_k^T}{y_k^T s_k}, \quad k = 0, 1, 2\dots, \tag{19}$$

and the search direction

$$d_{k+1} = -\frac{\|s_k\|}{\|y_k\|}F_{k+1} + \frac{\|s_k\|}{\|y_k\|}\frac{F_{k+1}^T y_k}{\|y_k\|^2}y_k - \frac{F_{k+1}^T s_k}{s_k^T y_k}s_k.$$

Now, trace of the matrix H_{k+1} in (19) can be computed as

$$\text{tr}(H_{k+1}) = \frac{\|s_k\|}{\|y_k\|}n - \frac{\|s_k\|}{\|y_k\|} + \frac{\|s_k\|^2}{s_k^T y_k}. \tag{20}$$

It can be seen that the third-term on the right of (20) will shift the eigenvalues of H_{k+1} towards the right making the matrix to have large eigenvalues. On the other hand, the second term being negative tends to shift the eigenvalues to the left. Even though this effect is countered by the first term, which is positive, it does not ensure proper distribution of the eigenvalues of H_{k+1}, and by extension stability of the scheme is not guaranteed.

Remark 2.2 *As explained above, while the choices of the parameters μ_k and γ_k in (13), (14), (16), and (17) may enable the scheme generated with the corresponding matrix H_{k+1} to satisfy the vital condition for analyzing global convergence, they do not ensure its stability – a condition attributed to uneven distribution of eigenvalues of H_{k+1}. In addition to this, like most of the published works in the literature for the constrained problem (1), the methods in [53–56] depend on the Lipschitz condition for proving their global convergence. And since not all problems satisfy the Lipschitz condition, the number of problems analyzed tend to be limited.*

Now, by replacing s_k with $\bar{y}_{k-1}$, y_k with F_{k-1}, B_{k+1} with Q_k in (6), and motivated by the scaling strategy in (10) and the above remark, we present the following two parameter scaled symmetric rank-two quasi-Newton type update,

$$Q_k = \frac{1}{\gamma_{k-1}}\left[Q_{k-1} - \frac{Q_{k-1}\bar{y}_{k-1}\bar{y}_{k-1}^T Q_{k-1}}{\bar{y}_{k-1}^T Q_{k-1}\bar{y}_{k-1}}\right] + \frac{1}{\delta_{k-1}}\frac{F_{k-1}F_{k-1}^T}{F_{k-1}^T\bar{y}_{k-1}}, \tag{21}$$

in which $F_{k-1} = F(x_{k-1})$ and

$$\bar{y}_{k-1} = F(\psi_{k-1}) - F(x_{k-1}) + \nu s_{k-1}, \quad \psi_{k-1} = x_{k-1} + \alpha_{k-1}d_{k-1}, \quad \nu > 0. \tag{22}$$

Setting Q_{k-1} as the identity matrix yields the following scaled memoryless version of (21):

$$Q_k = \frac{1}{\gamma_{k-1}}\left[I - \frac{\bar{y}_{k-1}\bar{y}_{k-1}^T}{\bar{y}_{k-1}^T\bar{y}_{k-1}}\right] + \frac{1}{\delta_{k-1}}\frac{F_{k-1}F_{k-1}^T}{F_{k-1}^T\bar{y}_{k-1}}. \tag{23}$$

Now, in order to define search direction of the proposed scheme, we require the inverse of the update Q_k and appropriate values for the positive parameters γ_{k-1} and δ_{k-1}. To achieve that goal, we first show that Q_k is positive-definite. Assuming that $F_{k-1}^T\bar{y}_{k-1} \geq m\|F_{k-1}\|\|\bar{y}_{k-1}\| > 0$, with $m \in (0,1]$, then we have that $F_{k-1} \neq 0$ and $\bar{y}_{k-1} \neq 0$. This further implies that a set of mutually orthonormal vectors $v_{k-1}^1, v_{k-1}^2, \ldots v_{k-1}^{n-2} \in \mathbb{R}^n$ exists such that $F_{k-1}^T v_{k-1}^i = \bar{y}_{k-1}^T v_{k-1}^i = 0$, $i = 1, \ldots, n-2$, which determines that

$$Q_k v_{k-1}^i = Q_k^T v_{k-1}^i = \frac{1}{\gamma_{k-1}}v_{k-1}^i, \quad i = 1, \ldots, n-2.$$

Thus, Q_k contains $n-2$ eigenvalues equal to $\frac{1}{\gamma_{k-1}}$ each. We label the two eigenvalues left as ζ_{k-1}^+ and ζ_{k-1}^-.

From (23), the trace of Q_k is obtained as

$$\text{tr}(Q_k) = \frac{n}{\gamma_{k-1}} - \frac{1}{\gamma_{k-1}} + \frac{\|F_{k-1}\|^2}{\delta_{k-1}F_{k-1}^T\bar{y}_{k-1}}$$
$$= \underbrace{\frac{1}{\gamma_{k-1}} + \ldots + \frac{1}{\gamma_{k-1}}}_{\text{(n-2) times}} + \zeta_{k-1}^{+} + \zeta_{k-1}^{-}, \tag{24}$$

which then reveals that

$$\zeta_{k-1}^{+} + \zeta_{k-1}^{-} = \frac{1}{\gamma_{k-1}} + \frac{\|F_{k-1}\|^2}{\delta_{k-1}F_{k-1}^T\bar{y}_{k-1}}. \tag{25}$$

Applying the Sherman-Morrison formula [36], the inverse of Q_k is computed as

$$Q_k^{-1} = \gamma_{k-1}I + \frac{(\delta_{k-1}F_{k-1}^T\bar{y}_{k-1} + \gamma_{k-1}\|F_{k-1}\|^2)\bar{y}_{k-1}\bar{y}_{k-1}^T}{(F_{k-1}^T\bar{y}_{k-1})^2} - \frac{\gamma_{k-1}\bar{y}_{k-1}F_{k-1}^T}{F_{k-1}^T\bar{y}_{k-1}} - \frac{\gamma_{k-1}F_{k-1}\bar{y}_{k-1}^T}{F_{k-1}^T\bar{y}_{k-1}}. \tag{26}$$

Since Q_k^{-1} has $n-2$ repeated eigenvalues equal to γ_{k-1} each, labelling the remaining two as $\frac{1}{\zeta_{k-1}^{+}}$ and $\frac{1}{\zeta_{k-1}^{-}}$, we obtain from (26) that

$$det(Q_k^{-1}) = \gamma_{k-1}^{(n-2)}\frac{1}{\zeta_{k-1}^{+}\zeta_{k-1}^{-}}. \tag{27}$$

Also, expressing Q_k^{-1} as the product of two matrices yields

$$Q_k^{-1} = \gamma_{k-1}I\left(I + \frac{(\delta_{k-1}F_{k-1}^T\bar{y}_{k-1} + \gamma_{k-1}\|F_{k-1}\|^2)\bar{y}_{k-1}\bar{y}_{k-1}^T}{\gamma_{k-1}(F_{k-1}^T\bar{y}_{k-1})^2} - \frac{\bar{y}_{k-1}F_{k-1}^T}{F_{k-1}^T\bar{y}_{k-1}} - \frac{F_{k-1}\bar{y}_{k-1}^T}{F_{k-1}^T\bar{y}_{k-1}}\right).$$

Let $A = \gamma_{k-1}I$ and $B = I + \frac{((\delta_{k-1}F_{k-1}^T\bar{y}_{k-1}+\gamma_{k-1}\|F_{k-1}\|^2)\bar{y}_{k-1}-\gamma_{k-1}(F_{k-1}^T\bar{y}_{k-1})F_{k-1})\bar{y}_{k-1}}{\gamma_{k-1}(F_{k-1}^T\bar{y}_{k-1})^2}$, then

$$det(A) = \gamma_{k-1}^{n}. \tag{28}$$

By applying the formula for determinant of an arbitrary matrix of rank two, namely

$$det(I + a_1a_2^T + a_3a_4^T) = (1 + a_1^Ta_2)(1 + a_3^Ta_4) - (a_1^Ta_4)(a_2^Ta_3),$$

and setting $a_1 = -\frac{\bar{y}_{k-1}}{F_{k-1}^T\bar{y}_{k-1}}$, $a_2 = F_{k-1}$, $a_3 = \frac{(\delta_{k-1}F_{k-1}^T\bar{y}_{k-1}+\gamma_{k-1}\|F_{k-1}\|^2)\bar{y}_{k-1}-\gamma_{k-1}(F_{k-1}^T\bar{y}_{k-1})F_{k-1}}{\gamma_{k-1}(F_{k-1}^T\bar{y}_{k-1})^2}$, and $a_4 = \bar{y}_{k-1}$, we obtain

$$det(B) = \frac{\delta_{k-1}\|\bar{y}_{k-1}\|^2}{\gamma_{k-1}F_{k-1}^T\bar{y}_{k-1}}. \tag{29}$$

Therefore, from (28) and (29), we obtain

$$det(Q_k^{-1}) = \gamma_{k-1}^{(n-1)}\frac{\delta_{k-1}\|\bar{y}_{k-1}\|^2}{F_{k-1}^T\bar{y}_{k-1}}. \tag{30}$$

Now, combining (27), (30) with some simplification, we get

$$\zeta_{k-1}^{+}\zeta_{k-1}^{-} = \frac{F_{k-1}^T \bar{y}_{k-1}}{\gamma_{k-1}\delta_{k-1}\|\bar{y}_{k-1}\|^2}. \tag{31}$$

Using (25) and (31), we obtain

$$\zeta_{k-1}^{\pm} = \frac{1}{2}\left[\frac{1}{\gamma_{k-1}} + \frac{\|F_{k-1}\|^2}{\delta_{k-1}F_{k-1}^T\bar{y}_{k-1}} \pm \sqrt{\left(\frac{1}{\gamma_{k-1}} + \frac{\|F_{k-1}\|^2}{\delta_{k-1}F_{k-1}^T\bar{y}_{k-1}}\right)^2 - 4\frac{F_{k-1}^T\bar{y}_{k-1}}{\gamma_{k-1}\delta_{k-1}\|\bar{y}_{k-1}\|^2}}\right],$$

which can also be expressed as

$$\zeta_{k-1}^{\pm} = \frac{1}{2}\left[\frac{1}{\gamma_{k-1}} + \frac{\|F_{k-1}\|^2}{\delta_{k-1}F_{k-1}^T\bar{y}_{k-1}} \right. \\ \left. \pm\sqrt{\left(\frac{1}{\gamma_{k-1}} - \frac{\|F_{k-1}\|^2}{\delta_{k-1}F_{k-1}^T\bar{y}_{k-1}}\right)^2 + 4\frac{\|F_{k-1}\|^2}{\gamma_{k-1}\delta_{k-1}F_{k-1}^T\bar{y}_{k-1}} - 4\frac{F_{k-1}^T\bar{y}_{k-1}}{\gamma_{k-1}\delta_{k-1}\|\bar{y}_{k-1}\|^2}}\right]. \tag{32}$$

Now, by applying the inequality in (11) to (32), we obtain

$$\begin{aligned}\zeta_{k-1}^{+} &\geq \frac{1}{2}\left[\frac{1}{\gamma_{k-1}} + \frac{\|F_{k-1}\|^2}{\delta_{k-1}F_{k-1}^T\bar{y}_{k-1}} + \sqrt{\left(\frac{1}{\gamma_{k-1}} - \frac{\|F_{k-1}\|^2}{\delta_{k-1}F_{k-1}^T\bar{y}_{k-1}}\right)^2}\right] \\ &= \frac{1}{2}\left[\frac{1}{\gamma_{k-1}} + \frac{\|F_{k-1}\|^2}{\delta_{k-1}F_{k-1}^T\bar{y}_{k-1}} + \frac{1}{\gamma_{k-1}} - \frac{\|F_{k-1}\|^2}{\delta_{k-1}F_{k-1}^T\bar{y}_{k-1}}\right] \\ &= \frac{1}{\gamma_{k-1}}.\end{aligned} \tag{33}$$

Also, by employing the Cauchy Schwarz inequality in (11) and applying similar argument, we have that $0 < \zeta_{k-1}^{-} \leq \frac{1}{\gamma_{k-1}}$, which applying (33) consequently yields

$$0 < \zeta_{k-1}^{-} \leq \frac{1}{\gamma_{k-1}} \leq \zeta_{k-1}^{+}. \tag{34}$$

Following (34), and since γ_{k-1} is a positive parameter, we conclude that the matrix Q_k is positive-definite, which implies that Q_k^{-1} is also positive-definite. We are now set to employ the measure function of Byrd and Nocedal [45] to compute approximations of γ_{k-1} and δ_{k-1} that will ensure well-conditioning of the matrix Q_k as well as better distribution of its eigenvalues. The measure function is formulated as

$$\phi(Q_k) = \text{tr}(Q_k) - \ln(det(Q_k)). \tag{35}$$

From (23) and applying the same approach employed to compute the determinant of Q_k^{-1} earlier, we obtain the determinant of Q_k as

$$det(Q_k) = \frac{\gamma_{k-1}^{(1-n)}}{\delta_{k-1}}\frac{F_{k-1}^T\bar{y}_{k-1}}{\|\bar{y}_{k-1}\|^2}. \tag{36}$$

Now, from (24), (35) and (36), we have

$$\begin{aligned}\phi(Q_k) &= \frac{n}{\gamma_{k-1}} - \frac{1}{\gamma_{k-1}} + \frac{\|F_{k-1}\|^2}{\delta_{k-1}F_{k-1}^T\bar{y}_{k-1}} - \ln\left(\frac{\gamma_{k-1}^{(1-n)}}{\delta_{k-1}}\frac{F_{k-1}^T\bar{y}_{k-1}}{\|\bar{y}_{k-1}\|^2}\right)\\ &= \frac{n}{\gamma_{k-1}} - \frac{1}{\gamma_{k-1}} + \frac{\|F_{k-1}\|^2}{\delta_{k-1}F_{k-1}^T\bar{y}_{k-1}} - \ln(\gamma_{k-1}^{(1-n)}) + \ln(\delta_{k-1}) - \ln(F_{k-1}^T\bar{y}_{k-1}) + \ln(\|\bar{y}_{k-1}\|^2)\\ &= \frac{n}{\gamma_{k-1}} - \frac{1}{\gamma_{k-1}} + \frac{\|F_{k-1}\|^2}{\delta_{k-1}F_{k-1}^T\bar{y}_{k-1}} - (1-n)\ln(\gamma_{k-1}) + \ln(\delta_{k-1}) - \ln(F_{k-1}^T\bar{y}_{k-1})\\ &+ \ln(\|\bar{y}_{k-1}\|^2).\end{aligned} \tag{37}$$

In order to obtain approximations for γ_{k-1} and δ_{k-1}, we have to minimize the function $\phi(Q_k)$ with respect to both γ_{k-1} and δ_{k-1}. So, from (37), we get

$$\frac{d\phi(Q_k)}{d\gamma_{k-1}} = \frac{1}{\gamma_{k-1}^2} - \frac{n}{\gamma_{k-1}^2} - \frac{(1-n)}{\gamma_{k-1}}.$$

Setting this result to zero, yields

$$\frac{1}{\gamma_{k-1}^2} - \frac{n}{\gamma_{k-1}^2} - \frac{(1-n)}{\gamma_{k-1}} = 0,$$

which implies that

$$\gamma_{k-1}(1-n) = 1-n,$$

or

$$\gamma_{k-1} = 1. \tag{38}$$

Also,

$$\frac{d\phi(Q_k)}{d\delta_{k-1}} = \frac{1}{\delta_{k-1}} - \frac{1}{\delta_{k-1}^2}\frac{\|F_{k-1}\|^2}{F_{k-1}^T\bar{y}_{k-1}}.$$

Setting this result to zero yields

$$\frac{1}{\delta_{k-1}} - \frac{1}{\delta_{k-1}^2}\frac{\|F_{k-1}\|^2}{F_{k-1}^T\bar{y}_{k-1}} = 0,$$

which implies that

$$\frac{1}{\delta_{k-1}^2}\frac{\|F_{k-1}\|^2}{F_{k-1}^T\bar{y}_{k-1}} = \frac{1}{\delta_{k-1}},$$

or

$$\delta_{k-1} = \frac{\|F_{k-1}\|^2}{F_{k-1}^T\bar{y}_{k-1}}. \tag{39}$$

Following the above analysis and the fact that restarting search directions tend to improve convergence and efficiency of algorithms [67], we substitute (38) and (39) into (26), and propose the following auxiliary search direction:

$$d_k = \begin{cases} -F_k, \quad k = 0, \\ -F_k + \frac{F_k^T F_{k-1}}{F_{k-1}^T \bar{y}_{k-1}} \bar{y}_{k-1} + \frac{F_k^T \bar{y}_{k-1}}{F_{k-1}^T \bar{y}_{k-1}} F_{k-1} - 2\frac{\|F_{k-1}\|^2 F_k^T \bar{y}_{k-1}}{(F_{k-1}^T \bar{y}_{k-1})^2} \bar{y}_{k-1}, \quad \text{for } k \geq 1 \text{ and} \\ F_{k-1}^T \bar{y}_{k-1} \geq m\|F_{k-1}\|\|\bar{y}_{k-1}\|, \quad m \in (0,1], \\ -F_k + \frac{1}{r}\frac{F_k^T \bar{y}_{k-1}}{\bar{y}_{k-1}^T \bar{y}_{k-1}} \bar{y}_{k-1} - \frac{|F_{k-1}^T \bar{y}_{k-1}|}{\|F_{k-1}\|\|\bar{y}_{k-1}\|} \frac{F_k^T F_{k-1}}{\|F_{k-1}\|^2} F_{k-1}, \quad r > 1, \quad \text{otherwise.} \end{cases} \tag{40}$$

Remark 2.3 *It should be noted that the inequality* $F_{k-1}^T \bar{y}_{k-1} \geq m\|F_{k-1}\|\|\bar{y}_{k-1}\|$, *which triggers the restart process was required for two reasons: one, it is essential for analyzing eigenvalues of the matrix* Q_k, *and two, it helps in proving boundedness of the search direction.*

Lemma 2.4 *The search direction* d_k *in (40) satisfies the inequality*

$$d_k^T F_k \leq c\|F_k\|^2, \quad c = \min\left\{\left(1 - \frac{1}{r}\right), \frac{1}{2}\right\}, \quad \forall k \geq 1. \tag{41}$$

Proof: First, from (40) for $k=0$, we have

$$d_0^T F_0 = -F_0^T(F_0) = -\|F_0\|^2.$$

Now, we proceed to analyze each of the two cases in (40) for $k \geq 1$.

1st Case: If $F_{k-1}^T \bar{y}_{k-1} \geq m\|F_{k-1}\|\|\bar{y}_{k-1}\|$, we have

$$\begin{aligned} d_k^T F_k &= -\|F_k\|^2 + 2\frac{(F_k^T F_{k-1}) F_k^T \bar{y}_{k-1}}{F_{k-1}^T \bar{y}_{k-1}} - 2\frac{\|F_{k-1}\|^2 (F_k^T \bar{y}_{k-1})^2}{(F_{k-1}^T \bar{y}_{k-1})^2} \\ &= \frac{2F_k^T F_{k-1}(F_{k-1}^T \bar{y}_{k-1}) F_k^T \bar{y}_{k-1} - (F_{k-1}^T \bar{y}_{k-1})^2 \|F_k\|^2 - 2\|F_{k-1}\|^2 (F_k^T \bar{y}_{k-1})^2}{(F_{k-1}^T \bar{y}_{k-1})^2} \\ &\leq \frac{\frac{\|F_k\|^2 (F_{k-1}^T \bar{y}_{k-1})^2}{2} + 2\|F_{k-1}\|^2 (F_k^T \bar{y}_{k-1})^2 - \|F_k\|^2 (F_{k-1}^T \bar{y}_{k-1})^2 - 2\|F_{k-1}\|^2 (F_k^T \bar{y}_{k-1})^2}{(F_{k-1}^T \bar{y}_{k-1})^2} \\ &= -\|F_k\|^2 + \frac{\|F_k\|^2}{2} \\ &= -\left(1 - \frac{1}{2}\right)\|F_k\|^2 \\ &= -\frac{1}{2}\|F_k\|^2. \end{aligned}$$

2nd Case: If $F_{k-1}^T \bar{y}_{k-1} < m\|F_{k-1}\|\|\bar{y}_{k-1}\|$, then from (40) and Cauchy Schwarz inequality, we get

$$\begin{aligned} d_k^T F_k &= -\|F_k\|^2 + \frac{1}{r}\frac{(F_k^T \bar{y}_{k-1})^2}{\bar{y}_{k-1}^T \bar{y}_{k-1}} - \frac{|F_{k-1}^T \bar{y}_{k-1}|}{\|F_{k-1}\|\|\bar{y}_{k-1}\|} \frac{(F_k^T F_{k-1})^2}{\|F_{k-1}\|^2} \\ &\leq -\|F_k\|^2 + \frac{1}{r}\frac{(F_k^T \bar{y}_{k-1})^2}{\bar{y}_{k-1}^T \bar{y}_{k-1}} \\ &\leq -\|F_k\|^2 + \frac{1}{r}\frac{\|F_k\|^2 \|\bar{y}_{k-1}\|^2}{\|\bar{y}_{k-1}\|^2} \\ &= -\|F_k\|^2 + \frac{1}{r}\|F_k\|^2 \\ &= -\left(1 - \frac{1}{r}\right)\|F_k\|^2. \end{aligned}$$

By setting $c = \min\left\{\left(1-\frac{1}{r}\right), \frac{1}{2}\right\}$, we established the proof. ■

For the line search strategy, we employ the adaptive procedure proposed by Yin et. al. [68], where the step-size α_k is defined as $\alpha_k = \beta\rho^{i_k}$, with i_k being the smallest nonnegative integer i for which

$$-F(\psi_k)^T d_k \geq \sigma\alpha_k P_{[\lambda,u]}[\|F(\psi_k)\|]\|d_k\|^2, \tag{42}$$

for each $u \in \mathbb{R}^n$, $P_{[\lambda,u]}[.]$ is the projection operator defined as

$$P_{[\lambda,u]}[h] = \begin{cases} u, & \text{if } h \geq u, \\ \lambda, & \text{if } h \leq \lambda, \\ h, & \text{otherwise,} \end{cases}$$

where $0 < \lambda \leq u$, and $u > 0$.

Now, we described algorithm of the method as follows:

Algorithm 1 (MKAS)

1: *Initialization*. Select $x_0 \in \mathcal{B}$, $\sigma > 0$, $\rho(0,1)$, $m \in (0,1]$, $r > 1$, $\beta \in (0,1]$, $\vartheta \in (0,2)$. Set $d_0 = -F_0$, $k = 0$.

2: For $\|F_k\| \leq 10^{-10}$, stop. Else, move to **3**.

3: Compute ψ_k as in (22), with α_k as defined earlier such that (42) holds.

4: If $\psi_k \in \mathcal{B}$ and $\|F(\psi_k)\| \leq 10^{-10}$, stop $x_{k+1} = \psi_k$. Otherwise, compute

$$x_{k+1} = P_{\mathcal{B}}\left[x_k - \vartheta\varsigma_k F(\psi_k)\right], \quad \text{where} \tag{43}$$

$P_{\mathcal{B}}(.)$ is the projection operator, which is defined in [69] with the property

$$\|P_{\mathcal{B}}(x) - y\| \leq \|x - y\|, \quad \forall y \in \mathcal{B}, \tag{44}$$

with

$$\varsigma_k = \frac{F(\psi_k)^T(x_k - \psi_k)}{\|F(\psi_k)\|^2}. \tag{45}$$

5: *Testing* $F_{k-1}^T\bar{y}_{k-1}$, $\|F_{k-1}\|$ *and* $\|\bar{y}_{k-1}\|$. Compute $F_{k-1}^T\bar{y}_{k-1}$, $\|F_{k-1}\|$ and $\|\bar{y}_{k-1}\|$.

6: Set $d_k = -F_k + \frac{F_k^T F_{k-1}}{F_{k-1}^T\bar{y}_{k-1}}\bar{y}_{k-1} + \frac{F_k^T\bar{y}_{k-1}}{F_{k-1}^T\bar{y}_{k-1}}F_{k-1} - 2\frac{\|F_{k-1}\|^2 F_k^T\bar{y}_{k-1}}{(F_{k-1}^T\bar{y}_{k-1})^2}\bar{y}_{k-1}$ if $F_{k-1}^T\bar{y}_{k-1} \geq m\|F_{k-1}\|\|\bar{y}_{k-1}\|$, where $m \in (0,1]$, otherwise compute $d_k = -F_k + \frac{F_k^T\bar{y}_{k-1}}{\bar{y}_{k-1}^T\bar{y}_{k-1}}\bar{y}_{k-1} - \frac{1}{r}\frac{|F_{k-1}^T\bar{y}_{k-1}|}{\|F_{k-1}\|\|\bar{y}_{k-1}\|}\frac{F_k^T F_{k-1}}{\|F_{k-1}\|^2}F_{k-1}$, where $r > 1$.

7: Set $k = k+1$. Goto **2**.

3 Analysis of convergence

We now proceed to analyze global convergence of the MKAS algorithm: First, we give the following assumptions:

Assumption 3.1 *The mapping F is monotone.*

Assumption 3.2 *There exists $\bar{x} \in \mathcal{B}$ such that $F(\bar{x}) = 0$.*

Lemma 3.3 *The search direction d_k generated in step 6 of the MKAS algorithm satisfy the inequality*

$$\min\left\{\left(1-\frac{1}{r}\right), \frac{1}{2}\right\}\|F_k\| \leq \|d_k\| \leq N\|F_k\|, \quad N = \left(1 + \frac{2}{m} + \frac{2}{m^2}\right). \tag{46}$$

Proof. By applying (41), Cauchy-Schwarz inequality (11), the first inequality is satisfied. Now, from (40), and for $k = 0$, we have that $d_0 = -F_0$, which shows that $\|d_0\| = \|F_0\|$. For $k \geq 1$, we consider the case of $F_{k-1}^T \bar{y}_{k-1} \geq m\|F_{k-1}\|\|\bar{y}_{k-1}\|$. From (40) and Cauchy-Schwarz inequality, we have

$$\begin{aligned}\|d_k\| &= \|-F_k + \frac{F_k^T F_{k-1}}{F_{k-1}^T \bar{y}_{k-1}}\bar{y}_{k-1} + \frac{F_k^T \bar{y}_{k-1}}{F_{k-1}^T \bar{y}_{k-1}}F_{k-1} - 2\frac{\|F_{k-1}\|^2 F_k^T \bar{y}_{k-1}}{(F_{k-1}^T \bar{y}_{k-1})^2}\bar{y}_{k-1}\| \\ &\leq \|F_k\| + \frac{\|F_k\|\|F_{k-1}\|\|\bar{y}_{k-1}\|}{F_{k-1}^T \bar{y}_{k-1}} + \frac{\|F_k\|\|F_{k-1}\|\|\bar{y}_{k-1}\|}{F_{k-1}^T \bar{y}_{k-1}} + 2\frac{\|F_{k-1}\|^2\|F_k\|\|\bar{y}_{k-1}\|^2}{(F_{k-1}^T \bar{y}_{k-1})^2} \\ &\leq \|F_k\| + \frac{\|F_k\|\|F_{k-1}\|\|\bar{y}_{k-1}\|}{m\|F_{k-1}\|\|\bar{y}_{k-1}\|} + \frac{\|F_k\|\|F_{k-1}\|\|\bar{y}_{k-1}\|}{m\|F_{k-1}\|\|\bar{y}_{k-1}\|} + 2\frac{\|F_{k-1}\|^2\|F_k\|\|\bar{y}_{k-1}\|^2}{(m\|F_{k-1}\|\|\bar{y}_{k-1}\|)^2} \\ &= \|F_k\| + \frac{\|F_k\|}{m} + \frac{\|F_k\|}{m} + 2\frac{\|F_k\|}{m^2} \\ &= \left(1 + \frac{2}{m} + \frac{2}{m^2}\right)\|F_k\|.\end{aligned}$$

Setting $N1 = \left(1 + \frac{2}{m} + \frac{2}{m^2}\right)$, we get

$$\|d_k\| \leq N1\|F_k\|.$$

For the second case, we have from (40) and Cauchy Schwarz inequality, that

$$\begin{aligned}\|d_k\| &= \left\|-F_k + \frac{1}{r}\frac{F_k^T \bar{y}_{k-1}}{\bar{y}_{k-1}^T \bar{y}_{k-1}}\bar{y}_{k-1} - \frac{|F_{k-1}^T \bar{y}_{k-1}|}{\|F_{k-1}\|\|\bar{y}_{k-1}\|}\frac{F_k^T F_{k-1}}{\|F_{k-1}\|^2}F_{k-1}\right\| \\ &\leq \|F_k\| + \frac{1}{r}\frac{\|F_k\|\|\bar{y}_{k-1}\|^2}{\|\bar{y}_{k-1}\|^2} + \frac{\|F_{k-1}\|^3\|F_k\|\|\bar{y}_{k-1}\|}{\|F_{k-1}\|^3\|\bar{y}_{k-1}\|} \\ &= \left(1 + \frac{1}{r} + 1\right)\|F_k\| \\ &= \left(2 + \frac{1}{r}\right)\|F_k\|.\end{aligned}$$

Setting $N2 = \left(2 + \frac{1}{r}\right)$, we get

$$\|d_k\| \leq N2\|F_k\|.$$

Therefore, since $N1$ is the max of $(1, N1, N2)$, we obtain that

$$\|d_k\| \leq \left(1 + \frac{2}{m} + \frac{2}{m^2}\right)\|F_k\|, \tag{47}$$

which proves that the second inequality of (46) holds. ∎

Lemma 3.4 *Given that F in (1) is continuous on* $\mathbb{R}^n$, *then for each* $k \geq 0$, *there exists nonnegative integer* i_k *such that (42) is satisfied.*

Proof. Suppose by contradiction that $k \geq 0$ exists for which (42) does not hold for any nonnegative integer i, i.e.,

$$\begin{aligned}-F(x_{k_0} + \beta\rho^i d_{k_0})^T d_{k_0} &< \sigma\beta\rho^i P_{[\lambda,u]}\|F(x_{k_0} + \beta\rho^i d_{k_0})\|\|d_{k_0}\|^2 \\ &\leq \sigma\beta\rho^i u\|d_{k_0}\|^2.\end{aligned} \tag{48}$$

The second inequality in (48) was obtained from the fact that $P_{[\lambda,u]}[.] \leq u$. By continuity of F, and letting $i \to \infty$ with $\rho \in (0,1)$, we get

$$F(x_{k_0})^T d_{k_0} \geq 0,$$

which contradicts

$$F(x_{k_0})^T d_{k_0} \leq -\min\left\{\left(1-\frac{1}{r}\right), \frac{1}{2}\right\}\|F_k\|^2 < 0.$$

Lemma 3.5 *Suppose Assumptions* 3.1 – 3.2 *hold and the sequences* $\{x_k\}$ *and* $\{\psi_k\}$ *are generated by Algorithm 1. Then for a solution* $\bar{x} \in \mathcal{B}$, *the sequence* $\{\|x_k - \bar{x}\|\}$ *is convergent and* $\{x_k\}$ *and* $\{\psi_k\}$ *are bounded.*

Furthermore

$$\lim_{k\to\infty} \alpha_k \|d_k\| = 0. \tag{49}$$

Proof: Using (42) and definition of ψ_k, we have

$$\begin{aligned} F(\psi_k)^T(x_k - \psi_k) &= -\alpha_k F(\psi_k)^T d_k \\ &\geq \sigma\alpha_k^2 P_{[\lambda,u]}\|F(\psi_k)\|\|d_k\|^2 \\ &\geq \sigma\lambda\alpha_k^2\|d_k\|^2 \\ &= \sigma\lambda\|x_k - \psi_k\|^2 > 0. \end{aligned} \tag{50}$$

By (2) and for all $\bar{x} \in \mathcal{B}$, we have

$$\begin{aligned} F(\psi_k)^T(x_k - \bar{x}) &= F(\psi_k)^T(x_k - \psi_k) + F(\psi_k)^T(\psi_k - \bar{x}) \\ &\geq F(\psi_k)^T(x_k - \psi_k) + F(\bar{x})^T(\psi_k - \bar{x}) \\ &= F(\psi_k)^T(x_k - \psi_k) \\ &\geq \sigma\lambda\|x_k - \psi_k\|^2 > 0. \end{aligned} \tag{51}$$

Combining the inequality (51) above, (43), (44) and (45), we obtain

$$\begin{aligned} \|x_{k+1} - \bar{x}\|^2 &= \|P_{\mathcal{B}}[x_k - \vartheta\varsigma_k F(\psi_k)] - \bar{x}\|^2 \\ &\leq \|x_k - \vartheta\varsigma_k F(\psi_k) - \bar{x}\|^2 \\ &= \|(x_k - \bar{x}) - \vartheta\varsigma_k F(\psi_k)\|^2 \\ &= \|x_k - \bar{x}\|^2 - 2\vartheta\varsigma_k F(\psi_k)^T(x_k - \bar{x}) + \vartheta^2\varsigma_k^2\|F(\psi_k)\|^2 \\ &\leq \|x_k - \bar{x}\|^2 - 2\vartheta\varsigma_k F(\psi_k)^T(x_k - \psi_k) + \vartheta^2\varsigma_k^2\|F(\psi_k)\|^2 \\ &= \|x_k - \bar{x}\|^2 - \vartheta(2-\vartheta)\frac{(F(\psi_k)^T(x_k - \psi_k))^2}{\|F(\psi_k)\|^2} \\ &\leq \|x_k - \bar{x}\|^2 - \vartheta(2-\vartheta)\frac{\sigma^2\lambda^2\|x_k - \psi_k\|^4}{\|F(\psi_k)\|^2}, \end{aligned} \tag{52}$$

where the last inequality indicates that $0 \leq \|x_{k+1} - \bar{x}\| \leq \|x_k - \bar{x}\|$ holds. The implication of this is that $\{\|x_k - \bar{x}\|\}$ is a monotone decreasing sequence that is bounded below. So, $\{\|x_k - \bar{x}\|\}$ is convergent and $\{x_k\}$ is bounded. Now, since $\{x_k\}$ is bounded and F is continuous, we see that there exists a constant $\bar{\vartheta} > 0$ such that for all $k \geq 0$,

$$\|x_k\| \leq \bar{\vartheta}, \quad \|F(x_k)\| \leq \bar{\vartheta}. \tag{53}$$

Combining (53) with (47) shows that $\{d_k\}$ is bounded. Furthermore, from (2), Cauchy Schwarz inequality, and (50), we have

$$\begin{aligned}\bar{\vartheta} \geq \|F_k\| &\geq \frac{F_k^T(x_k - \psi_k)}{\|x_k - \psi_k\|} \geq \frac{F(\psi_k)^T(x_k - \psi_k)}{\|x_k - \psi_k\|} \geq \sigma\lambda\|x_k - \psi_k\| \\ &\geq \sigma\lambda\|\psi_k\| - \sigma\lambda\bar{\vartheta},\end{aligned}$$

which further yields

$$\|\psi_k\| \leq \frac{\bar{\vartheta} + \sigma\lambda\bar{\vartheta}}{\sigma\lambda},$$

which clearly shows the boundedness of $\{\psi_k\}$, which by continuity of F, indicates boundedness of $\{F(\psi_k)\}$, namely, a constant κ exists for which

$$\|F(\psi_k)\| \leq \kappa. \ \ \forall k \geq 0.$$

Combining this result with the last inequality of (52) yields

$$\begin{aligned}\vartheta(2-\vartheta)\frac{\sigma^2\lambda^2}{\kappa^2}\sum_{k=0}^{\infty}\|x_k - \psi_k\|^4 &\leq \sum_{k=0}^{\infty}(\|x_k - \bar{x}\|^2 - \|x_{k+1} - \bar{x}\|^2) \\ &= \|x_0 - \bar{x}\|^2 - \lim_{k\to\infty}\|x_{k+1} - \bar{x}\|^2 < \infty,\end{aligned}$$

which further implies that $0 \leq \lim_{k\to\infty}\|x_k - \psi_k\| = \lim_{k\to\infty}\alpha_k\|d_k\|$. Hence the proof is obtained. ■

Theorem 3.6 *The sequence $\{x_k\}$ generated by MKAS Algorithm converges globally to a solution of (1).*

Proof. Two cases are analyzed for the proof

1st case: Assuming that $\liminf_{k\to 0}\|F_k\| = 0$, then the boundedness of $\{x_k\}$ and continuity of F implies that some cluster points $\hat{x}$ exists for which $F(\hat{x}) = 0$. Also, since $\{\|x_k - \hat{x}\|\}$ is convergent, it implies that $\{x_k\}$ converges to $\hat{x}$.

2nd case: $\liminf_{k\to 0}\|F_k\| > 0$. In this case we have that a constant $\bar{c} > 0$ exists such that

$$\|F_k\| \geq \bar{c}, \quad \forall k \geq 0. \tag{54}$$

From first inequality in (46), we have

$$\|d_k\| \geq \min\left\{\left(1 - \frac{1}{r}\right), \frac{1}{2}\right\}\|F_k\| \geq \min\left\{\left(1 - \frac{1}{r}\right), \frac{1}{2}\right\}\bar{c}.$$

Combining this result with (49) yields

$$\lim_{k\to\infty} \alpha_k = 0. \tag{55}$$

By the boundedness of the sequences $\{x_k\}$ and $\{d_k\}$, we see that there exists subsequences $\{x_{k_i}\}$ and $\{d_{k_i}\}$ for which

$$\lim_{i\to\infty, i\in\mathcal{K}} x_{k_i} = \hat{x}, \quad \lim_{i\to\infty, i\in\mathcal{K}} d_{k_i} = \hat{d},$$

with $\mathcal{K}$ being an infinite indexing set. From (46), we get

$$-F(x_{k_i})^T d_{k_i} \geq \min\left\{\left(1-\frac{1}{r}\right), \frac{1}{2}\right\} \|F(x_{k_i})\|^2, \quad \forall i \in \mathcal{K}. \tag{56}$$

Letting $i \to \infty$ in (56) with continuity of F and (54), we obtain

$$-F(\hat{x})^T \hat{d} \geq \min\left\{\left(1-\frac{1}{r}\right), \frac{1}{2}\right\} \|F(\hat{x})\|^2 > \min\left\{\left(1-\frac{1}{r}\right), \frac{1}{2}\right\} \bar{c}^2 > 0. \tag{57}$$

On the other hand, from (42), if $\alpha_k \neq \beta$, then $\bar{\alpha} = \frac{\alpha}{\rho}$ will not satisfy (42), i.e.,

$$\begin{aligned} -F(x_{k_i} + \rho^{-1}\alpha_{k_i} d_{k_i})^T d_{k_i} &< \sigma\rho^{-1}\alpha_{k_i} P_{[\lambda,u]}[\|F(x_{k_i} + \rho^{-1}\alpha_{k_i} d_{k_i})\|]\|d_{k_i}\|^2 \\ &\leq \sigma\rho^{-1}\alpha_{k_i} u \|d_{k_i}\|^2 \end{aligned} \tag{58}$$

By taking limit as $i \to \infty$, with $i \in \mathcal{K}$ in (58), (55) and continuity of F, we obtain

$$-F(\hat{x})^T \hat{d} \leq 0,$$

which shows a contradiction with (57). Hence the proof is established. ■

4 Numerical experiments

To test the MKAS method's effectiveness, it is compared with the QN-type methods in [55] labelled SMDFP, [57] labelled SMBFGS and [56] labelled PBDFP. To code the four algorithms, MATLAB *R*2014*a* was employed with a PC configured as (2.30ghz cpu, 4GB RAM). The criteria for stopping the iterations is $\|F(x_k)\| \leq 10^{-10}$ or $\|F(\psi_k)\| \leq 10^{-10}$ or iterations exceed 1000 without attaining a solution. Parameters values for MKAS are set as $\beta = 1$, $\rho = 0.5$, $\sigma = 0.01$, $\vartheta = 1.99$, $\nu = 0.001$, $m = 0.01$, $r = 1.6$, $\lambda = 0.001$ and $u = 0.8$. Parameter values for the other algorithms are set according to how they are used in the papers.

The following test examples with dimensions 1000, 5000, and 10000, where F is given as: $F = (f_1(x), f_2(x), ..., f_n(x))^T$.

Example 1 This is the gradient of the strictly convex test example in [70] where $\mathcal{B} = \mathbb{R}^n_+$ added to yield
$f_i(x) = \exp x_i - 1, \quad i = 1, 2, \ldots, n.$
Example 2 This is a modified version of the test example in [71] with $\mathcal{B} = \mathbb{R}^n_+$ added to yield
$f_1(x) = \exp x_1 - 1,$
$f_i(x) = \exp x_i + x_i - 1, \quad i = 2, \ldots, n.$
Example 3 [72] with $\mathcal{B} = \mathbb{R}^n_+$
$f_i(x) = 2x_i - \sin|x_i|, \quad i = 1, 2, \ldots, n.$
Example 4 [73].
$f_1(x) = x_1 - \exp\left(cos\left(\frac{x_1+x_2}{2}\right)\right),$

$f_i(x) = x_i - \exp\left(cos\left(\frac{x_{i-1}+x_i+x_{i+1}}{i}\right)\right), \quad i = 2, 3, \ldots, n-1,$
$f_n(x) = x_n - \exp\left(cos\left(\frac{x_{n-1}+x_n}{n}\right)\right),$
with $\mathcal{B} = \mathbb{R}^n_+$.
Example 5 A modified version of the test example in [72] with $\mathcal{B} = \mathbb{R}^n_+$
$f_i(x) = 3x_i - \sin x_i, \quad i = 1, 2, \ldots, n.$
Example 6 A modification of the test example 3 with $\mathcal{B} = \mathbb{R}^n_+$
$f_i(x) = 3x_i + \cos x_i - 1, \quad i = 1, 2, \ldots, n.$
Example 7 A modification of the test example 3 with $\mathcal{B} = \mathbb{R}^n_+$ added to yield
$f_1(x) = 2x_1 + \exp\sin x_1 - 1,$
$f_i(x) = 2x_{i-1} + \exp\sin x_i + 2x_i - 1,$
$f_n(x) = 2x_n + \exp\sin x_n - 1, \; i = 2, \ldots, n-1.$
Example 8 A modification of the test example 3 with $\mathcal{B} = \mathbb{R}^n_+$ added to yield
$f_1(x) = \sin x_1 + \exp\sin x_1 - 1,$
$f_i(x) = \sin x_i + \exp\sin x_i + x_i - 1, \quad i = 2, \ldots, n.$
Example 9 A modification of the test example 2 with $\mathcal{B} = \mathbb{R}^n_+$ added to yield
$f_1(x) = 3x_1 + \exp\sin x_1 - 1,$
$f_i(x) = 3x_i + \exp\sin x_i - 1, \; i = 2, \ldots, n.$
The following initial guesses were used:
$\boldsymbol{x}_1 = \left(\frac{1}{n}, \frac{2}{n}, \ldots, 1\right)^T$, $\boldsymbol{x}_2 = \left(1, \frac{1}{2}, \ldots, \frac{1}{n}\right)^T$, $\boldsymbol{x}_3 = \left(\frac{1}{2}, \frac{3}{2}, \ldots, -\frac{[(-1)^n-2]}{2}\right)^T$, $\boldsymbol{x}_4 = (1, 1, \ldots, \ldots, 1)^T$,
$\boldsymbol{x}_5 = \left(\frac{3}{4}, \frac{1}{4}, \ldots, -\frac{[(-1)^n-2]}{4}\right)^T$, $\boldsymbol{x}_6 = (2, 2, \ldots, 2)^T$. The numerical result of the experiments for the four algorithms is displayed in three tables, and is accessible from the link https://github.com/hungugida/hungugida/blob/main/Tables1-3MKAS.pdf, where "N" and "Pdim" denotes example of the operator F and dimension, "SP" and "Nit" represents for initial guess and number of iterations. Also, "Fval," "Ptime(s)" and "Norm" stands for function evaluations, processing time and the residual at stopping point, while "***" denotes failure to obtain a solution.

In order describe the values shown in the above link, we use the evaluation too by Dolan and Moré [74]. Figs 1–3 were plotted for iterations, function evaluations, and CPU time performance metrics. From Fig 1, we see that MKAS solved about 95% of the test examples with less number of iterations. This values however, include 9% solved by the algorithm together with PBDFP, SMDFP, and SMBFGS. It also showed that SMDFP solved 15% of the test examples with less iterations. Here also, this value includes 12% solved with the other algorithms. Fig 2 showed that MKAS solved 93% of the test examples with less function evaluations. It also showed that PBDFP solved 8%, which includes 2% solved with SMDFP, and SMBFGS algorithms. For least CPU time metric, Fig 3 showed that MKAS perform better than PBDFP, SMDFP, and SMBFGS because it solved 89% while the latter recorded 8%, 1% and 2%. To further validate the results of the experiments, we present the average residual obtained for each of the four algorithms as computed from tables in the above link as follows: MKAS (1.21×10^{-11}), PBDFP (4.41×10^{-11}), SMDFP (1.83×10^{-10}) and SMBFGS (1.75×10^{-10}). Hence, the above analysis indicates that MKAS method is better suited for solving (1) than the other schemes.

5 Concluding remarks and future research

We have presented a new quasi-Newton-type method for solving constrained system of monotone nonlinear equations in this article. The method is an adaptive modification of the BFGS method for unconstrained optimization. The scheme possesses the vital property for analyzing global convergence as well as the trust region property irrespective of the line search procedure; what's more, the global convergence was proven without the Lipschitz condition, thereby broadening its applicability to a wider class of problems. Another important finding was the impact of the restart strategy in improving

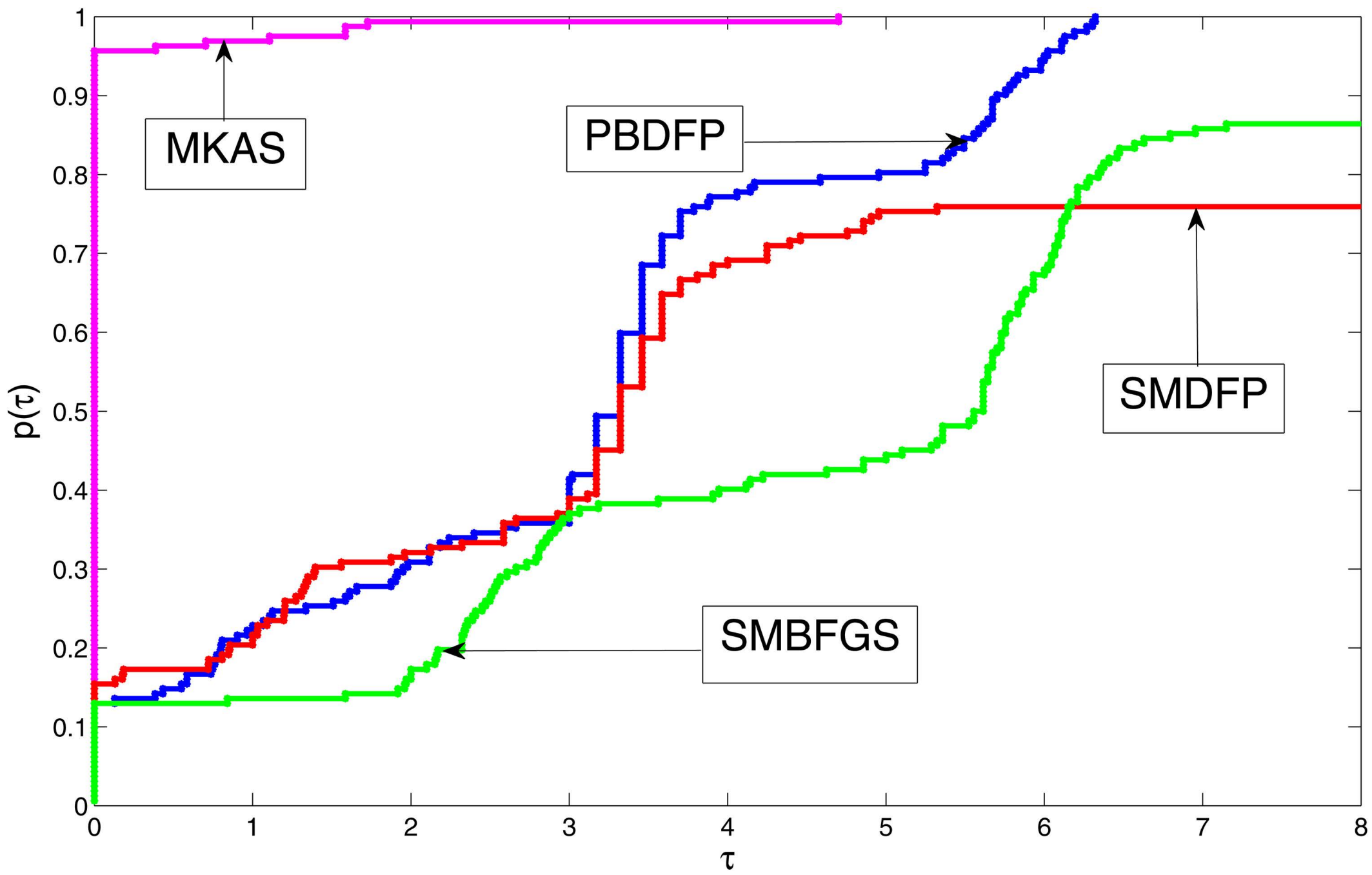

Fig 1. Performance profile of the four methods for number of iterations.

https://doi.org/10.1371/journal.pone.0344697.g001

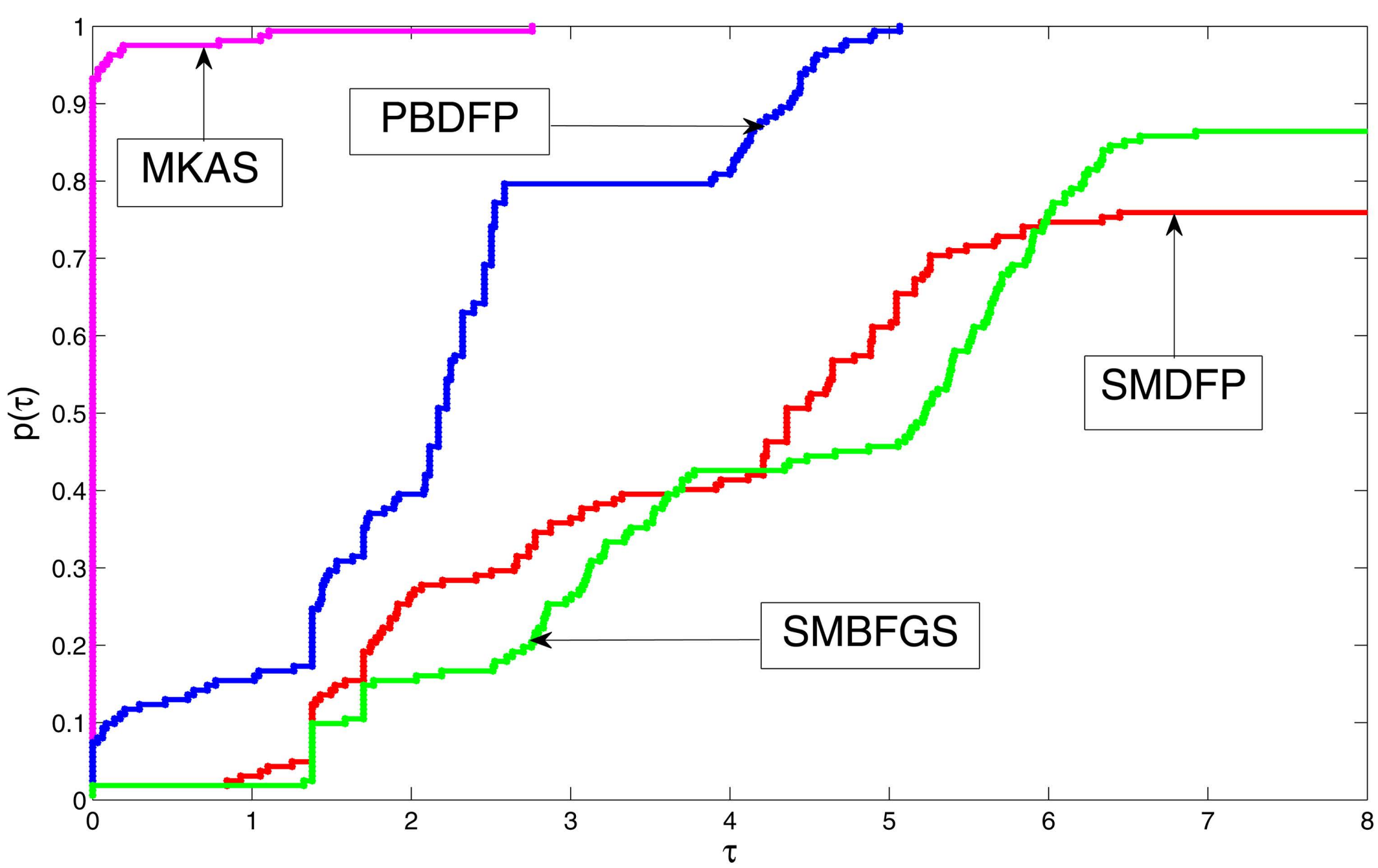

Fig 2. Performance profile of the four methods for function evaluations.

https://doi.org/10.1371/journal.pone.0344697.g002

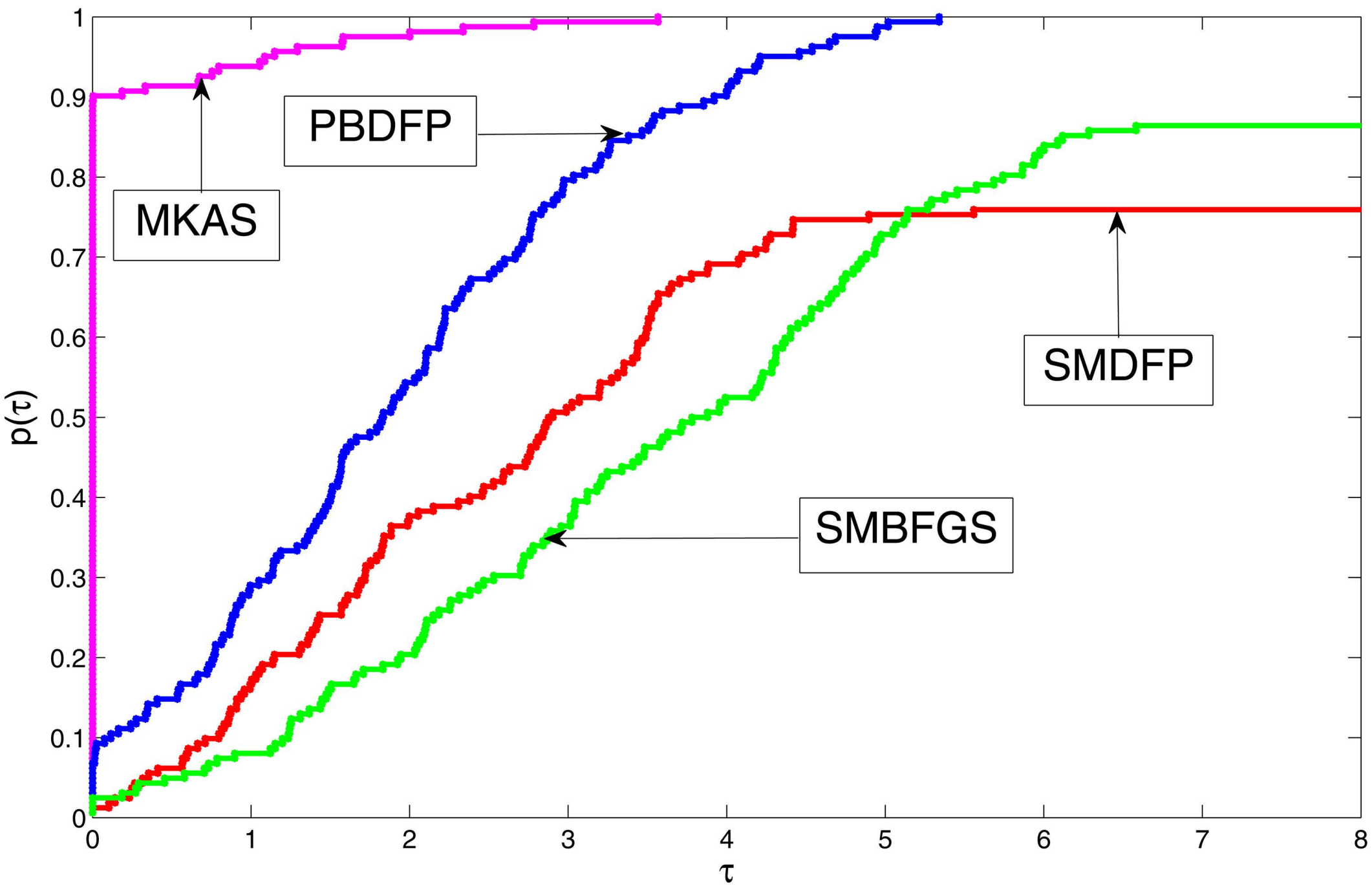

Fig 3. Performance profile of the four methods for CPU time.

https://doi.org/10.1371/journal.pone.0344697.g003

convergence of the scheme, as evident in the best average residual it recorded. Furthermore, numerical experiments with the method and some of the available quasi-Newton type methods for solving constrained system of monotone nonlinear equations showed that it is effective. The focus of a future research will be to develop a variant of the proposed method, where the global convergence will be analyzed without the monotonicity and Lipschitz assumptions. We also hope to include real-life applications of the method in the study.

Author contributions

Conceptualization: Kabiru Ahmed, Abubakar Sani Halilu.

Data curation: Salisu Murtala.

Formal analysis: Kabiru Ahmed, Abubakar Sani Halilu.

Funding acquisition: Hatem E Semary.

Investigation: Kabiru Ahmed, Muhammed Yusuf Waziri.

Methodology: Kabiru Ahmed, Abubakar Sani Halilu.

Project administration: Asmaa S. Al-Moisheer, Muhammed Yusuf Waziri, Mohamad Afendee Mohamed.

Resources: Hatem E Semary, Asmaa S. Al-Moisheer, Salisu Murtala.

Software: Sulaiman M. Ibrahim, Salisu Murtala.

Supervision: Hatem E Semary, Muhammed Yusuf Waziri, Mohamad Afendee Mohamed.

Validation: Hatem E Semary, Asmaa S. Al-Moisheer, Muhammed Yusuf Waziri, Mohamad Afendee Mohamed, Salisu Murtala.

Visualization: Sulaiman M. Ibrahim.

Writing – original draft: Kabiru Ahmed, Abubakar Sani Halilu.

Writing – review & editing: Hatem E Semary, Asmaa S. Al-Moisheer, Sulaiman M. Ibrahim, Mohamad Afendee Mohamed, Salisu Murtala.

References

1. Dirkse SP, Ferris MC. Mcplib: a collection of nonlinear mixed complementarity problems. Optimization Methods and Software. 1995;5(4):319–45. https://doi.org/10.1080/10556789508805619
2. Meintjes K, Morgan AP. A methodology for solving chemical equilibrium systems. Applied Mathematics and Computation. 1987;22(4):333–61. https://doi.org/10.1016/0096-3003(87)90076-2
3. Prajna S, Parrilo PA, Rantzer A. Nonlinear Control Synthesis by Convex Optimization. IEEE Trans Automat Contr. 2004;49(2):310–4. https://doi.org/10.1109/tac.2003.823000
4. Luo ZQ, Yui W. An introduction to convex optimization for communications and signal processing. IEEE J Select Areas Commun. 2006;24(8):1426–38. https://doi.org/10.1109/jsac.2006.879347
5. Evgeniou T, Pontil M, Toubia O. A Convex Optimization Approach to Modeling Consumer Heterogeneity in Conjoint Estimation. Marketing Science. 2007;26(6):805–18. https://doi.org/10.1287/mksc.1070.0291
6. Xiao Y, Zhu H. A conjugate gradient method to solve convex constrained monotone equations with applications in compressive sensing. Journal of Mathematical Analysis and Applications. 2013;405(1):310–9. https://doi.org/10.1016/j.jmaa.2013.04.017
7. Halilu AS, Majumder A, Waziri MY, Awwal AM, Ahmed K. On solving double direction methods for convex constrained monotone nonlinear equations with image restoration. Comp Appl Math. 2021;40(7). https://doi.org/10.1007/s40314-021-01624-1
8. Ahmed K, Waziri MY, Halilu AS, Murtala S. On two symmetric Dai-Kou type schemes for constrained monotone equations with image recovery application. EURO Journal on Computational Optimization. 2023;11:100057. https://doi.org/10.1016/j.ejco.2023.100057
9. Ahmed K, Waziri MY, Murtala S, Halilu AS, Sabi'u J. On a Scaled Symmetric Dai–Liao-Type Scheme for Constrained System of Nonlinear Equations with Applications. J Optim Theory Appl. 2023;200(2):669–702. https://doi.org/10.1007/s10957-023-02281-6
10. Ahmed K, Waziri MY, Halilu AS, Murtala S, Sabi'u J. Another Hager-Zhang-type method via singular-value study for constrained monotone equations with application. Numer Algor. 2023;96(4):1583–623. https://doi.org/10.1007/s11075-023-01678-8
11. Ahmed K, Waziri MY, Halilu AS, Murtala S, Abdullahi H. Signal and image reconstruction with a double parameter Hager–Zhang-type conjugate gradient method for system of nonlinear equations. Numerical Linear Algebra App. 2024;32(1). https://doi.org/10.1002/nla.2583
12. Ahmed K, Waziri MY, Sani Halilu A, Murtala S. Two RMIL-type schemes with compressed sensing applications. Optimization Methods and Software. 2024;40(3):455–86. https://doi.org/10.1080/10556788.2024.2425001
13. Kiri AI, Waziri MY, Ahmed K. A modified Liu-Storey scheme for nonlinear systems with an application to image recovery. Iranian Journal of Numerical Analysis and Optimization. 2023;13:1–21.
14. Ahmed K, Yusuf Waziri M, Sani Halilu A, Murtala S. Sparse signal reconstruction via Hager–Zhang-type schemes for constrained system of nonlinear equations. Optimization. 2023;73(6):1949–80. https://doi.org/10.1080/02331934.2023.2187255
15. Solodov MV, Svaiter BF. A Globally Convergent Inexact Newton Method for Systems of Monotone Equations. Applied Optimization. Springer US. 1998. 355–69. https://doi.org/10.1007/978-1-4757-6388-1_18
16. Broyden CG. A class of methods for solving nonlinear simultaneous equations. Math Comp. 1965;19(92):577–93. https://doi.org/10.1090/s0025-5718-1965-0198670-6
17. Ortega JM, Rheinboldt WC. Iterative solution of nonlinear equations in several variables. New York: Academic Press. 1970.
18. Dennis JE, Schnabel RB. Numerical Methods for Unconstrained Optimization and Nonlinear Equations. Englewood Cliffs, NJ: Prentice-Hall. 1983.
19. Gao P, He C. An efficient three-term conjugate gradient method for nonlinear monotone equations with convex constraints. Calcolo. 2018;55(4). https://doi.org/10.1007/s10092-018-0291-2
20. Halilu AS, Majumder A, Waziri MY, Ahmed K, Awwal AM. Motion control of the two joint planar robotic manipulators through accelerated Dai–Liao method for solving system of nonlinear equations. EC. 2021;39(5):1802–40. https://doi.org/10.1108/ec-06-2021-0317
21. Halilu AS, Majumder A, Waziri MY, Ahmed K. Signal recovery with convex constrained nonlinear monotone equations through conjugate gradient hybrid approach. Mathematics and Computers in Simulation. 2021;187:520–39. https://doi.org/10.1016/j.matcom.2021.03.020

22. Koorapetse M, Kaelo P, Lekoko S, Diphofu T. A derivative-free RMIL conjugate gradient projection method for convex constrained nonlinear monotone equations with applications in compressive sensing. Applied Numerical Mathematics. 2021;165:431–41. https://doi.org/10.1016/j.apnum.2021.03.005
23. Liu JK, Li SJ. A projection method for convex constrained monotone nonlinear equations with applications. Computers & Mathematics with Applications. 2015;70(10):2442–53. https://doi.org/10.1016/j.camwa.2015.09.014
24. Liu J, Feng Y. A derivative-free iterative method for nonlinear monotone equations with convex constraints. Numer Algor. 2018;82(1):245–62. https://doi.org/10.1007/s11075-018-0603-2
25. Kanzow C, Yamashita N, Fukushima M. Levenberg–Marquardt methods with strong local convergence properties for solving nonlinear equations with convex constraints. Journal of Computational and Applied Mathematics. 2004;172(2):375–97. https://doi.org/10.1016/j.cam.2004.02.013
26. Bellavia S, Macconi M, Morini B. STRSCNE: A Scaled Trust-Region Solver for Constrained Nonlinear Equations. Computational Optimization and Applications. 2004;28(1):31–50. https://doi.org/10.1023/b:coap.0000018878.95983.4e
27. Liu J, Li S. Multivariate spectral DY-type projection method for convex constrained nonlinear monotone equations. Journal of Industrial & Management Optimization. 2017;13(1):283–95. https://doi.org/10.3934/jimo.2016017
28. Yu G, Niu S, Ma J. Multivariate spectral gradient projection method for nonlinear monotone equations with convex constraints. Journal of Industrial & Management Optimization. 2013;9(1):117–29. https://doi.org/10.3934/jimo.2013.9.117
29. Davidon WC. Variable metric method for minimization. Argonne, IL: Argonne National Laboratory. 1959.
30. Fletcher R, Powell MJD. A Rapidly Convergent Descent Method for Minimization. The Computer Journal. 1963;6(2):163–8. https://doi.org/10.1093/comjnl/6.2.163
31. Sun W, Yuan YX. Optimization Theory and Methods: Nonlinear Programming. New York: Springer. 2006.
32. Broyden CG. The convergence of a class of double-rank minimization algorithms. IMA Journal of Applied Mathematics. 1970;6:76–90.
33. Fletcher R. A new approach to variable metric algorithms. The Computer Journal. 1970;13(3):317–22. https://doi.org/10.1093/comjnl/13.3.317
34. Goldfarb D. A family of variable-metric methods derived by variational means. Math Comp. 1970;24(109):23–6. https://doi.org/10.1090/s0025-5718-1970-0258249-6
35. Shanno DF. Conditioning of quasi-Newton methods for function minimization. Math Comp. 1970;24(111):647–56. https://doi.org/10.1090/s0025-5718-1970-0274029-x
36. Sun W, Yuan YX. Optimization Theory and Methods: Nonlinear Programming. New York: Springer. 2006.
37. Andrei N. Eigenvalues versus singular values study in conjugate gradient algorithms for large-scale unconstrained optimization. Optimization Methods and Software. 2016;32(3):534–51. https://doi.org/10.1080/10556788.2016.1225211
38. Powell MJD. Some global convergence properties of a variable metric algorithm for minimization without exact line search. In: Cottle RW, Lemke CE, editors. Nonlinear Programming. Philadelphia: SIAM. 1976. 53–72.
39. Byrd RH, Liu DC, Nocedal J. On the Behavior of Broyden's Class of Quasi-Newton Methods. SIAM J Optim. 1992;2(4):533–57. https://doi.org/10.1137/0802026
40. Gill PE, Leonard MW. Reduced-Hessian Quasi-Newton Methods for Unconstrained Optimization. SIAM J Optim. 2001;12(1):209–37. https://doi.org/10.1137/s1052623400307950
41. Biggs MC. Minimization Algorithms Making Use of Non-quadratic Properties of the Objective Function. IMA J Appl Math. 1972;9(2):123–123. https://doi.org/10.1093/imamat/9.2.123
42. Oren SS, Luenberger DG. Self-scaling variable metric algorithms, Part I: Criteria and sufficient conditions for scaling a class of algorithms. Management Science. 1974;20:845–62.
43. Andrei N. A double parameter scaled BFGS method for unconstrained optimization. Journal of Computational and Applied Mathematics. 2018;332:26–44. https://doi.org/10.1016/j.cam.2017.10.009
44. Andrei N. A Double-Parameter Scaling Broyden–Fletcher–Goldfarb–Shanno Method Based on Minimizing the Measure Function of Byrd and Nocedal for Unconstrained Optimization. J Optim Theory Appl. 2018;178(1):191–218. https://doi.org/10.1007/s10957-018-1288-3
45. Byrd RH, Nocedal J. A Tool for the Analysis of Quasi-Newton Methods with Application to Unconstrained Minimization. SIAM J Numer Anal. 1989;26(3):727–39. https://doi.org/10.1137/0726042
46. Babaie-Kafaki S. A hybrid scaling parameter for the scaled memoryless BFGS method based on the ℓ_∞ matrix norm. International Journal of Computer Mathematics. 2018;96(8):1595–602. https://doi.org/10.1080/00207160.2018.1465940
47. Babaie–Kafaki S, Aminifard Z. Two–parameter scaled memoryless BFGS methods with a nonmonotone choice for the initial step length. Numer Algor. 2019;82(4):1345–57. https://doi.org/10.1007/s11075-019-00658-1
48. Aminifard Z, Babaie-Kafaki S. Analysis of the Maximum Magnification by the Scaled Memoryless DFP Updating Formula with Application to Compressive Sensing. Mediterr J Math. 2021;18(6). https://doi.org/10.1007/s00009-021-01905-3
49. Dargahi F, Babaie-Kafaki S, Aminifard Z. Eigenvalue Analyses on the Memoryless Davidon–Fletcher–Powell Method Based on a Spectral Secant Equation. J Optim Theory Appl. 2023;200(1):394–403. https://doi.org/10.1007/s10957-023-02354-6

50. Bakhshinejad A, Babaie–Kafaki S. An extended version of the memoryless DFP algorithm with the sufficient descent property. Boll Unione Mat Ital. 2024;17(4):747–58. https://doi.org/10.1007/s40574-024-00408-9
51. Mamat M, Dauda MK, Mohamed MA, Waziri MY, Mohamad FS, Abdullah H. Derivative free Davidon-Fletcher-Powell (DFP) for solving symmetric systems of nonlinear equations. IOP Conf Ser: Mater Sci Eng. 2018;332:012030. https://doi.org/10.1088/1757-899x/332/1/012030
52. Dauda MK. Modified Conjugate Gradient Method via DFP Update for Solving Systems of Nonlinear Equations. myjcam. 2020;3(1):30–6. https://doi.org/10.37231/myjcam.2020.3.1.42
53. Awwal AM, Kumam P, Sitthithakerngkiet K, Bakoji AM, Halilu AS. Derivative-free method based on DFP updating formula for solving convex constrained nonlinear monotone equations and application. AIMS Mathematics. 2021;6:8792–814.
54. Rao J, Huang N. A derivative-free scaling memoryless DFP method for solving large scale nonlinear monotone equations. J Glob Optim. 2022;87(2–4):641–77. https://doi.org/10.1007/s10898-022-01215-2
55. Ullah N, Shah A, Sabi'u J, Jiao X, Awwal AM. A one-parameter memoryless DFP algorithm for solving system of monotone nonlinear equations with application in image processing. Mathematics. 2023;11:1–26.
56. ur Rehman M, Sabi'u J, Sohaib M, Shah A. A projection-based derivative free DFP approach for solving system of nonlinear convex constrained monotone equations with image restoration applications. J Appl Math Comput. 2023;69(5):3645–73. https://doi.org/10.1007/s12190-023-01897-1
57. Shah A, ur Rehman M, Sabi'u J, Sohaib M, Furati KM. A new scaled BFGS method for convex constraints monotone systems: Applications in motion control. Results in Applied Mathematics. 2025;25:100531. https://doi.org/10.1016/j.rinam.2024.100531
58. Liu P, Li J, Shao H, Wu T. An accelerated derivative-free memoryless Davidon-Fletcher-Powell method and its iteration-complexity analysis. J Sci Comput. 2025;105(2). https://doi.org/10.1007/s10915-025-03071-0
59. Liu P, Li J, Shao H, Shao F, Liu M. An accelerated Dai–Yuan conjugate gradient projection method with the optimal choice. Engineering Optimization. 2025;57(12):3648–76. https://doi.org/10.1080/0305215x.2025.2450683
60. Liu P, Wu X, Shao H, Zhang Y, Cao S. Three adaptive hybrid derivative-free projection methods for constrained monotone nonlinear equations and their applications. Numerical Linear Algebra with Applications. 2023;30:e2471.
61. Liu P, Li L, Shao H, Liu M, Fan J. An Inertial-type CG Projection Method with Restart for Pseudo-monotone Costs with Application to Traffic Assignment. Netw Spat Econ. 2024;25(1):147–72. https://doi.org/10.1007/s11067-024-09653-z
62. Rehman M ur, Mahmood S, Sabi'u J, Sohaib M, Shah A, Furati KM. An efficient scaled DFP algorithm based on singular value analysis for solving systems of monotone nonlinear equations. Franklin Open. 2025;13:100422. https://doi.org/10.1016/j.fraope.2025.100422
63. Sabi'u J, Saksurakan C, Neamprem K, Sirisubtawee S. An Improved DFP Method for Solving Convex Constrained Monotone Equations with Application in Image Restoration. Int J Comput Methods. 2025;23(02). https://doi.org/10.1142/s0219876225500379
64. Sabi'u J, Shah A. An efficient three-term conjugate gradient-type algorithm for monotone nonlinear equations. RAIRO-Oper Res. 2021;55:S1113–27. https://doi.org/10.1051/ro/2020061
65. Sabi'u J, Shah A, Stanimirović PS, Ivanov B, Waziri MY. Modified optimal Perry conjugate gradient method for solving system of monotone equations with applications. Applied Numerical Mathematics. 2023;184:431–45. https://doi.org/10.1016/j.apnum.2022.10.016
66. Nocedal J, Wright SJ. Numerical Optimization. New York: Springer. 2006.
67. Powell MJD. Restart procedures for the conjugate gradient method. Mathematical Programming. 1977;12(1):241–54. https://doi.org/10.1007/bf01593790
68. Yin J, Jian J, Jiang X, Liu M, Wang L. A hybrid three-term conjugate gradient projection method for constrained nonlinear monotone equations with applications. Numer Algor. 2021;88(1):389–418. https://doi.org/10.1007/s11075-020-01043-z
69. Zarantonello EH. Projections on convex sets in Hilbert space and spectral theory. New York: Academic Press. 1971.
70. Raydan M. The Barzilai and Borwein Gradient Method for the Large Scale Unconstrained Minimization Problem. SIAM J Optim. 1997;7(1):26–33. https://doi.org/10.1137/s1052623494266365
71. Cruz WL, Martinez JM, Raydan M. Spectral residual method without gradient information for solving large-scale nonlinear systems of equations: theory and experiments. 2004.
72. Cruz WL. A spectral algorithm for large-scale systems of nonlinear monotone equations. Numer Algor. 2017;76(4):1109–30. https://doi.org/10.1007/s11075-017-0299-8
73. La Cruz W, Martínez J, Raydan M. Spectral residual method without gradient information for solving large-scale nonlinear systems of equations. Math Comp. 2006;75(255):1429–48. https://doi.org/10.1090/s0025-5718-06-01840-0
74. Dolan ED, Moré JJ. Benchmarking optimization software with performance profiles. Mathematical Programming. 2002;91(2):201–13. https://doi.org/10.1007/s101070100263
